# From waste to food: Optimising the breakdown of oil palm waste to provide substrate for insects farmed as animal feed

**DOI:** 10.1371/journal.pone.0224771

**Published:** 2019-11-07

**Authors:** Elizabeth Dickinson, Mark Harrison, Marc Parker, Michael Dickinson, James Donarski, Adrian Charlton, Rosie Nolan, Aida Rafat, Florence Gschwend, Jason Hallett, Maureen Wakefield, Julie Wilson

**Affiliations:** 1 Department of Mathematics, University of York, Heslington, York, United Kingdom; 2 Fera Science Ltd., Sand Hutton, York, United Kingdom; 3 Biorenewables Development Centre Ltd, Dunnington, York, United Kingdom; 4 Department of Chemical Engineering, Imperial College London, London, United Kingdom; Gifu University, JAPAN

## Abstract

Waste biomass from the palm oil industry is currently burned as a means of disposal and solutions are required to reduce the environmental impact. Whilst some waste biomass can be recycled to provide green energy such as biogas, this investigation aimed to optimise experimental conditions for recycling palm waste into substrate for insects, farmed as a sustainable high-protein animal feed. NMR spectroscopy and LC-HRMS were used to analyse the composition of palm empty fruit bunches (EFB) under experimental conditions optimised to produce nutritious substrate rather than biogas. Statistical pattern recognition techniques were used to investigate differences in composition for various combinations of pre-processing and anaerobic digestion (AD) methods. Pre-processing methods included steaming, pressure cooking, composting, microwaving, and breaking down the EFB using ionic liquids. AD conditions which were modified in combination with pre-processing methods were ratios of EFB:digestate and pH. Results show that the selection of pre-processing method affects the breakdown of the palm waste and subsequently the substrate composition and biogas production. Although large-scale insect feeding trials will be required to determine nutritional content, we found that conditions can be optimised to recycle palm waste for the production of substrate for insect rearing. Pre-processing EFB using ionic liquid before AD at pH6 with a 2:1 digestate:EFB ratio were found to be the best combination of experimental conditions.

## Introduction

Lignocellulosic plant biomass is the most abundant raw material on Earth, composed of approximately 40–50% cellulose, 20–40% hemicellulose and 20–35% lignin [1]. In particular, the Oil Palm Industry, with plantations spreading across Asia, Africa and Latin America, creates vast waste streams made up of empty fruit bunches (EFB), oil palm fronds and trunks, the disposal of which is a major environmental problem, often burnt as a means of disposal, resulting in air pollution [2]. Agricultural waste of Peninsular Malaysia has been estimated at 17 Mt, 77% of which is from the oil palm industry [3]. Solutions of waste disposal are being sought, and some of those proposed are to use the waste palm kernel shells and palm press fibres as biofuel to create steam used in the oil palm mill processes, or the creation of mulch from EFB and ash to be reused as fertiliser, both of which lead to self-sufficiency, sustainability and improved net energy balance of the oil palm mills [2, 4]. Returning EFB to the land, however, can result in eutrophication [5], therefore, with no specific utilization of EFB, other breakdown technologies are being investigated, such as gasification for power generation [5, 6]. Waste biomass is also increasingly being used as a source of renewable energy in the production of gases such as methane by anaerobic digestion (AD) [7]. The high sugar content and abundance of lignocellulose also make it a promising substrate for microorganism cultivation and procedures such as AD and composting have the potential to produce highly nutritious substrate for insect growth [8]. In May 2017, the EU amended their regulations, allowing the feeding of insects to farmed fish largely as a high protein alternative to fishmeal [9], a positive step towards food sustainability that will help to address over fishing as demand for meat and fish increases. Moreover, insect protein provides the amino acids methionine and cysteine, essential to animal nutrition, but lacking in many plant based protein sources which are currently used in animal feed, such as soybean [10].

However, using insects reared on oil palm waste as a sustainable source of protein for farmed animals poses several challenges. EFB are intrinsically resistant to breakdown and processing must be optimised to produce the most nutritious substrate for larval growth. As a waste product which is naturally (but slowly) decomposed by fungi present in the local environment [11], the biomass requires mycotoxin monitoring, to ensure both larvae survival and that any toxins are not passed further up the food chain [12]. The use of AD to break down biomass, rather than fermentation or composting, should minimise the growth of fungi and thereby minimise the risk of mycotoxin contamination. AD is also a reproducible, low cost method, which can be established is oil palm mills of developing countries.

Here we evaluate the results of various pre-processing methods, designed to breakdown the biomass before AD, using metabolomic analysis with liquid chromatography–high resolution mass spectrometry (LC-HRMS) and nuclear magnetic resonance (NMR) spectroscopy. These techniques in combination with multivariate statistical methods allow detailed analysis of the biomass composition [13].

Microwaving at high temperature and pressure has been shown to be an effective method for initial biomass breakdown [14]. Gentler pre-processing methods such as steaming [15, 16] and heating under pressure may be more suitable, however, particularly in developing countries, as these processes will be less expensive than microwaving, and heat and steam are already generated and readily available within the oil palm mills [2, 4]. Ionic liquids are increasingly used for biomass breakdown [17], with the current focus on inexpensive and recoverable solvents [18].

Previous studies on the breakdown of EFB by AD (often mixed with other oil palm industry waste) focus on conditions that favour the production of biogas [16, 19, 20]. To the best of our knowledge, this is the first investigation of procedures (pre-processing and AD experimental conditions) that aims to minimise biogas production in order to retain nutrients and produce a substrate suitable for insect rearing. Conditions should favour proliferation of hydrolytic, acidogenic and acetogenic microorganisms, and minimise the growth of colonies of methanogenic microorganisms such as archaea [19] (Table 1). It is essential to establish the point at which AD should be stopped before production of toxic breakdown products. Small-scale experiments were carried out to allow various experimental conditions to be investigated in an iterative process and consequently the quantities of substrate produced were not adequate for an extensive feeding trial.

**Table 1:** Key stages during anaerobic digestion.

	AD Stage			
	Hydrolysis	Acidogenesis	Acetogenesis	Methanogenesis
Breakdown products and metabolites	Sugars Amino acids Fatty Acids	Volatile Fatty Acids Alcohols Carbon Dioxide Hydrogen Hydrogen sulphide	Acetate Hydrogen	Methane Carbon dioxide

Although conditions are optimised for larval feed, the biogases produced during AD are also studied here as these could potentially fuel the processing with any additional waste from the AD reused as fertiliser [7]. Efficient optimisation of biomass breakdown and substrate production could therefore have impact on farming policy and the possibility of energy-neutral sites.

## Materials and methods

**Fig 1:**
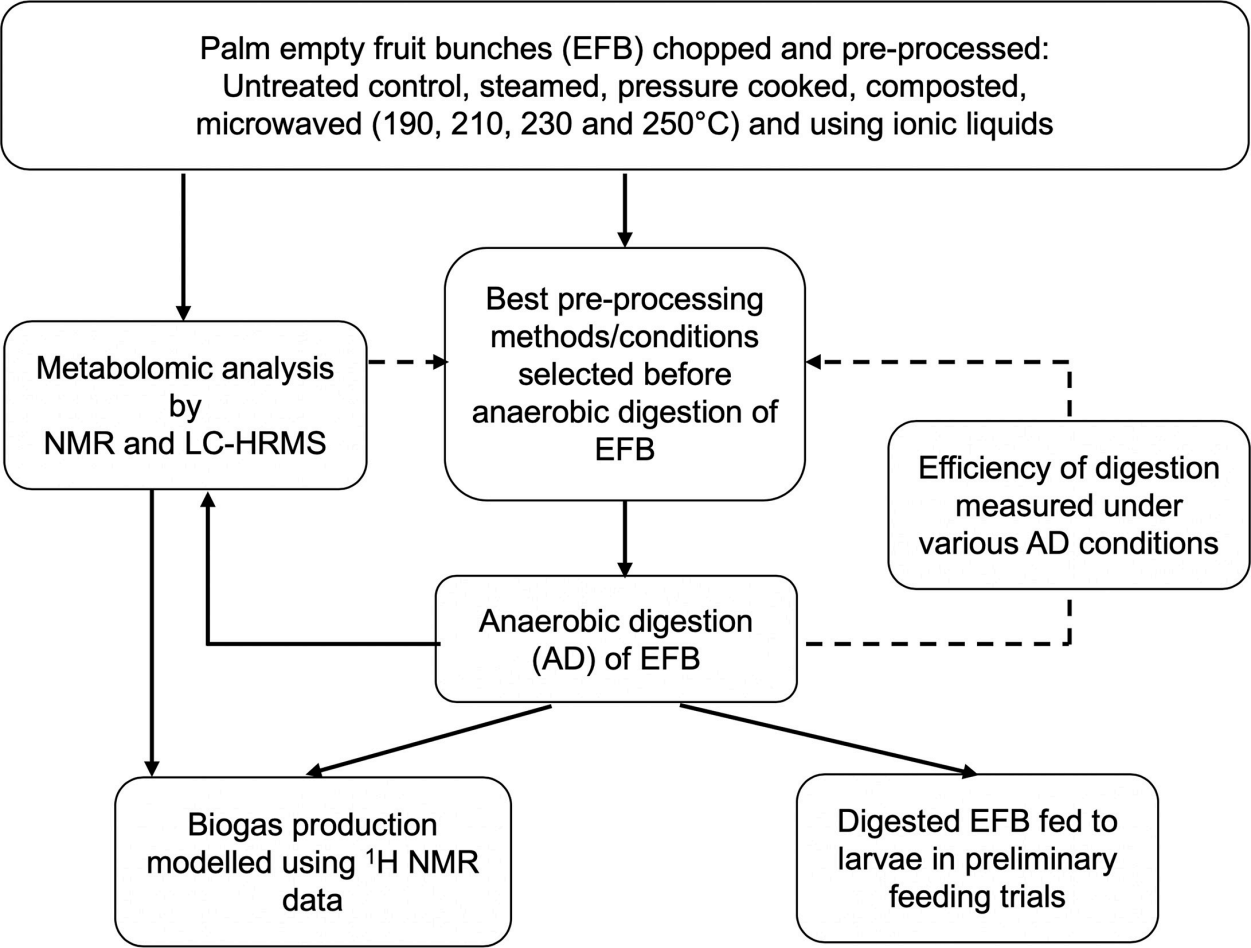
Flow–chart showing the iterative processes in the investigation. Results from metabolomic analyses were used to select suitable pre–processing methods, with a feedback loop for selection of AD experimental conditions. Only preliminary feeding trials could be carried out due to the small volumes of digested EFB produced.

The EFB was obtained from a sustainable, government owned, oil palm mill in Indonesia. The metabolomic analysis of pre-processed EFB was used to select the most appropriate methods prior to AD. AD conditions were optimised using a “feedback loop” (Fig 1). For clarity, pre-processed EFB will refer to samples prior to AD and digested EFB will refer to EFB samples post AD.

### EFB pre-processing

Secateurs were used to chop EFB into pieces of approximately 1cm in length and 100g reserved without further processing as a control. A 100g of the EFB were ground more finely using an analytical mill, referred to as “milled”. A further 100g of EFB was combined with 250ml of water in the rice bowl of a conventional kitchen steamer and steamed for 60 minutes, referred to as “steamed”.

For pressure cooked samples, 100g of EFB was placed on the trivet of a conventional kitchen hob pressure cooker and 250ml of water added, before cooking under pressure for 15 minutes. Due to concerns about the age of the original pressure cooker, this process was repeated with a new pressure cooker, giving 6 observations.

For each of four different temperatures (190, 210, 230 and 250°C), three vessels containing 3g of EFB and 60ml of water were microwaved separately (ramp = 15 min, hold = 0 min, maximum power = 1800W) and the pre-processed EFB combined from the three vessels. For 250°C, the pressure limit of 45 bar was reached and the temperature recorded as 247°C.

Although traditional composting process of EFB can take months or even years [21], short-term composting was investigated for comparison and to investigate possible fungal toxins. Chopped EFB were composted over a three-month period, by leaving on soil in a standard greenhouse, covered by black plastic. Due to the time taken for composting, analysis of these samples was not performed before AD.

N,N-dimethylbutylammonium hydrogen sulfate (synthesised in house) containing 20 wt% water was used for pre-processing EFB by ionic liquid (IL) [18] with 148g biomass (10 wt% moisture content) fed to 2L batch reactor with jacketed oil circulator. The preheated IL (950g) was added and the reaction time started when the internal temperature of the biomass and IL reached 150°C. Stirring was applied at 120 rpm and, after 1 hour reaction time, the oil bath was turned off and the reaction slurry allowed to cool down. When the temperature of the reaction slurry reached 75°C, the reactor was opened and the slurry transferred to a clean plastic container; ethanol was used to assist the transfer of the viscous slurry. The slurry/ethanol mix was then vacuum filtered to separate the cellulose pulp from the IL/ethanol. Washing with ethanol continued until a total of 2L had been added, before the cellulose pulp was transferred to four centrifuge falcon tubes. Water was added to each falcon tube, vortexed for 2 mins and then allowed to settle for 1 hour. This washing step was repeated four times, until wash water reached pH 5, to remove all IL.

### Metabolomics

EFB control, pre-processed and digested EFB samples were snap frozen in liquid nitrogen for 30 minutes then lyophilised for a minimum of 72 hours, before grinding. For both LC-HRMS and NMR, samples were analysed in triplicate to check for technical variation and the same extraction, sample preparation and data acquisition procedures were used for pre-processed EFB and digested EFB. For method evaluation, additional replicates were analysed for steamed EFB (six in total) and a further 230°C microwaved sample was analysed when acquiring 2D NMR data at a later date. Table 2 summarises the samples analysed.

**Table 2:** Details of sample conditions (ratios shown are digestate:EFB).

Pre-processed (before AD)	Digested EFBs (after AD)
Microwave 190°C Microwave 210°C Microwave 230°C Microwave 250°C Steamed Pressure Cooked Composted Ionic Liquids Milled Untreated Control	Microwave 2:1 pH 7 Pressure cooked 2:1 pH 7 Steamed 2:1 pH 7 Composted 2:1 pH 7 Ionic liquid 2:1 pH 7 Untreated Control 2:1 pH 7 Milled 2:1 pH 7
	Microwave 1:2 pH 7 Steamed 1:2 pH 7 Untreated Control 1:2 pH 7
	Microwave 2:1 pH 6 Pressure cooked 2:1 pH 6 Steamed 2:1 pH 6 Composted 2:1 pH 6 Ionic liquid 2:1 pH 6 Untreated Control 2:1 pH 6
	Digestate only pH7

Digestate was from an AD plant fed with maize silage and pig slurry. All metabolomic analyses were performed in triplicate.

#### LC-HRMS data acquisition and confirmatory analysis

75mg of lyophilised sample were extracted into 1.5ml 1:1 MeOH:H_2_O, shaken for 30 minutes, then centrifuged at 21,000*g* for 10min at 20°C. The supernatant (unfiltered) was then diluted with 1:1 MeOH: H_2_O and 1ml pipetted to 2ml screw top vials. Quality Control samples (QCs) for pre-processed EFB and for digested EFB were obtained by pooling the relevant EFB samples.

LC–HRMS analysis was conducted on samples in a random order, with QC samples run between every 8 experimental samples (a “batch”), resulting in four batches for pre-processed EFB samples and six batches for digested EFB, ensuring that no batch was dominated by any particular experimental group. A reverse phase aqueous chromatography column, ACE 3Q 150x3mm, 3μm (Advanced Chromatography Technologies, Aberdeen, UK), was used. Mobile phases were 0.1% formic acid in water (mobile phase A, MPA) and 0.1% formic acid in acetonitrile (mobile phase B, MPB). Gradient applied was 100% MPA for 5 minutes before increasing to 100% MPB over 15 minutes. This was held for 10 minutes before reverting to 100% MPA and held for 2 minutes. Injection volume was 10 μl, flow rate was 0.4 mlmin^-1^ and column temperature was 25°C. The MS used was a Thermo Exactive Orbitrap (Thermo Fisher Scientific, MA, USA.) set at 50,000 resolution FWHM @ 200 *m/z* with an acquisition speed of 2Hz. Data were acquired between 50 and 1000 *m/z* for 30 minutes per sample. The column was conditioned before sample analysis using 6 QC injections. Both positive and negative mode LC–HRMS data were acquired as two separate analytical runs for both pre-processed EFB and digested EFB samples, resulting in four LC-HRMS datasets (Table 3). Data alignment and peak picking were performed using Progenesis QI (Nonlinear Dynamics, Waters Corporation, Newcastle Upon Tyne, UK).

**Table 3:** The number of samples in the six data sets analysed with the number of peaks or data points found in each.

Pre-processed EFBs (before AD)	No of Samples	No of Peaks/Data points
LC-HRMS negative mode	35 (30 + 5 QCs)	1108
LC-HRMS positive mode	35 (30 + 5 QCs)	2881
NMR	31	31363
EFB Digestate (after AD)		
LC-HRMS negative mode	55 (48 + 7 QCs)	2181
LC-HRMS positive mode	55 (48 + 7 QCs)	2610
NMR	66	29816

LC–MS data were batch–corrected and peaks close to zero were removed before scaling. The water region was excluded from NMR data and, after AD, peaks related to acetic acid region were removed.

Confirmatory analyses were performed on an Orbitrap Velos Pro (Thermo Fisher Scientific, MA, USA.) A precursor list of potentially significant accurate masses was generated from the multivariate statistical analysis and used as precursor ions for MS/MS and MSn analysis in both positive and negative ionisation modes. Collision Induced Dissociation (CID) fragmentation was employed using 35 ev. A fragmentation event was only triggered if the precursor mass had a signal of 1000 (au) or greater. After fragmentation, FTMS detection was used to obtain accurate mass product ions. A MS^3^ event was subsequently triggered on the 3 most abundant product ions of the precursor mass if the product ions had a signal of 500 (au) or greater. MS^3^ ions were detected by the ion trap producing nominal mass product ions. Where available, corresponding analytical standards were analysed concurrently to confirm compound identity. LC conditions were as described above.

Sixteen analytical standards (≥97% purity, sourced from Sigma Aldrich, Gillingham, UK, TCI UK Ltd, Oxford, UK, Fisher Scientific UK Ltd, Loughborough, UK, and VWR International Ltd, Lutterworth, UK) were made to a stock concentration of 1mgml^-1^ in either methanol or ultra-pure water (18.2 MΩ), depending on solubility. A combined standard comprising all 16 metabolites at a concentration of 10μgml^-1^ in MeOH:H_2_O was produced and a dilution of this standard to 1μgml^-1^ also created. These standards were used to confirm/disconfirm the identity of the proposed 16 significant metabolites using retention time and product ion comparison (Table 4) [22].

**Table 4:** Metabolites responsible for variation in PCA of LC–HRMS data, identified using analytical standards and metabolite databases [17].

Mode	Principal Component	Metabolite [molecular species]	*m/z*	Retention Time (RT)	Level of ID
Pos	1	Syringaldehyde [M+H]^+^	183.065216	13.18	2
Pos	1	Levoglucosan [M+H]^+^	163.060043	2.09	1
Pos	1	Fusaric Acid [M+H]^+^	180.101898	2.09	1
Pos	2	Disaccharide [M+Na]^+^	365.105530	1.58	2
Neg	1	Xylose [M-H]^-^	149.044907	2.23	2
Neg	1	Glucose [M-H_2_O-H]^-^	161.044998	2.24	1
Neg	1	Malic Acid [M-H_2_O-H]^-^	115.002899	5.16	1
Neg	1	Vanillin [M-H]^-^	151.039398	13.44	2

#### NMR spectroscopy

75 mg of lyophilised sample were extracted in 1.5ml of 250mM D_2_O phosphate buffer (K_2_HPO_4_/KH_2_PO_4_, pH = 7.0) with 1mM sodium azide and 1mM trimethylsilylpropionic acid- d_4_ sodium salt (TSP), and vortexed for 30min. Samples were then centrifuged at 21,000*g* for 10min at 20°C. The supernatant was filtered using 13mm PTFE 0.45μm Klarity syringe filters, and 600μl added to 5mm Wilmad NMR tubes.

^1^H-NMR data was acquired on a Bruker Avance 500 MHz NMR spectrometer equipped with a TCI cryoprobe. 1D Spectra were acquired at a central frequency of 500.1323546 MHz data, for 256 transients (and 8 dummy scans) into 65536 data points over a spectral width of 14.0019 ppm, with 90° pulse length of 11.25 μs, with on-resonance pre-saturation to suppress the intensity of the water signal for 3s, and with a total delay of 6 s, giving an acquisition time of 4.67 s and a total experiment time of approximately 47 minutes. A sine bell-shaped window function phase shifted by 90° was applied over all data points before Fourier transformation, phase, and baseline correction. All spectra were acquired at 300 K. The chemical shift of all data was referenced to the TSP resonance at 0 ppm. In house software written for Matlab (The Mathworks, Inc, Natick, MA, USA) was used to produce a matrix of potential metabolites for each EFB sample (observation).

To aid metabolite assignment, 2D NMR data were acquired for some samples (Pre-processed EFB: IL and 230°C Microwave; Digested EFB: Pressure cooked, 230°C Microwave, and IL (all at 2:1 ratio straw digestate inoculant:EFB)). All data acquisition was performed at a temperature of 300 K without sample rotation, using the software package Topspin v 1.3 Patch level 3 (Bruker, Germany). ^1^H-^1^H total correlation spectroscopy (TOCSY) data were recorded using the standard spectrometer mlevphpr library pulse sequence (RD-90°_*x*_ *–t*_1_- mlev17-acq) with 16 unrecorded (dummy) scans and 8 transients over a spectral width of 14.0019ppm, into 4096 data points collected in F2 and 384 increments in F1, and TPPI phase cycling, with 90° pulse length of 11.25 μs, a *t*_1_ delay of 60.24μs and spin lock time of 150ms. On-resonance pre-saturation was used to suppress the intensity of the water signal during relaxation delay for 1.5s. Prior to Fourier transformation, a QSINE window function with Sine bell shift = 2 was applied in both dimensions, and data were zero filled to 4096 and 1024 data points for F2 and F1 respectively.

The ^1^H–^13^C gradient enhanced heteronuclear single quantum coherence (HSQC) NMR spectra were acquired using the standard library gradient enhanced pulse sequence [23] at the central frequencies of 500.1323541 MHz (^1^H, F2 dimension) and 125.7691082 MHz (^13^C, F1 dimension). A carbon–proton coupling constant of 145 Hz, gradient ratios, all using a sine shaped gradient of 80, 20.1, 11 and −5% for gradients 1, 2, 3 and 4, respectively, a homospoil gradient pulse length of 1 ms, a gradient pulse length of 600 μs, a homospoil gradient pulse recovery delay of 200 μs, a delay of 862.07 μs to select for all carbon proton multiplicities, an interscan delay of 1.5 s, and an acquisition mode of echo–antiecho were used. 90° pulse lengths of 11.25 and 13.0 μs were used for ^1^H and ^13^C, respectively, 1536 complex data points were collected in the F2 dimension with a spectral width of 13.33 ppm, with 8 unrecorded (dummy) scans and 8 transients, giving an acquisition time of 0.11532 s and proton carbon decoupling using a waltz decoupling sequence with a decoupling power of 10 dB was applied during the acquisition period. 512 increments were collected in the F1 dimension over a spectral width of 200.0 ppm. A QSINE window function with Sine bell shift = 2 was applied over all data points and the data were zero filled to 2048 and 1024 data points in the F2 and F1 dimensions, respectively, prior to Fourier transformation, phase and baseline correction. The chemical shifts of all data were referenced to the resonance of the TSP peak at 0 ppm in both the ^1^H and the ^13^C dimensions.

#### Statistical analysis

All data analyses were conducted in R version 3.4.4 (R Core Team 2018, R Foundation for Statistical Computing, Vienna, Austria). For LC-HRMS data, initial data exploration using principal component analysis (PCA) showed batch differences to be the greatest source of variance for each dataset (S1 Fig and S2 Fig). To remove this technical variation, batch correction techniques were applied [24]. The effectiveness of batch correction was assessed using the Bhattacharrya distance [25] (S1 Table) and F-tests to measure within/between batch variability. For 1D NMR, the spectral region for the water resonance was removed to prevent varying water suppression affecting analyses. In addition, as acetic acid was added to some digestion experiments to reduce the pH to 6 (see Methods Section and S2 Table), the region corresponding to acetic acid (1.90–1.94ppm) was removed from 1D NMR spectra obtained for digested EFB. Data were normalised to total sum of the spectral integral and UV-scaled data were used throughout analysis of NMR and LC-HRMS data, but unscaled data were used to check peak size.

PCA was performed on 1D NMR and positive and negative mode LC-HRMS data from both pre-processed and digested EFB, giving six data sets in total (Table 3).

Partial least squares regression (PLSR) was used to predict biogas production, using 1D NMR data from digested EFB and the plsr package [26]. All models were cross validated using 10-fold cross validation, or split into a training and test set (0.75:0.25), overfitting was checked by root mean square error of prediction (RMSEP) and by R^2^.

### Anaerobic Digestion (AD)

AD treatments were carried out at the Biorenewables Development Centre, York, using a biomethane potential kit (BMP) on all pre-processed EFB samples and untreated control, automatically measuring the cumulative biogas produced. Three experimental conditions were investigated (in triplicate) in each of three separate experimental runs over 21 days with the results from earlier runs used to inform later experiments (Fig 1). A volatile solids (VS)-based dosing ratio of one part digestate VS to two parts EFB feedstock VS was used in order to produce a more textured end product for larvae to attach to and compared with the standard 2:1 ratio and a pH value of 6 (in comparison to pH 7, both still at 2:1 ratio) was used to inhibit methanogenic microorganisms and favour hydrolytic, acidogenic and acetogenic microorganisms. The percentage of total dry solids and VS within the samples were determined by heating a known amount of sample at 105°C and 550°C, respectively, in a Thermolyne muffle furnace (Thermo Scientific, UK) until a steady weight was reached. BMP analysis (in triplicate) was carried out using an AER 800 respirometer (Challenge Technology, Arkansas, USA according to the manufacturer’s instructions [27]). The seed microbial culture/inocolum (digestate from an AD plant fed with maize silage and pig slurry) was sieved to remove any large particles to give a solution containing particulates that were < 1 mm. Exact quantities used in the BMP vessels to achieve the VS ratios are shown in S2 Table. For those experiments run at pH6, the initial pH was set to 6 using 5M acetic acid. Vessel headspace was purged with nitrogen to remove any residual air which may have affected the results. The BMP test was run under anaerobic conditions, at 35°C and 300 rpm. To prevent possible inhibition of larval growth due to the presence of metabolites in the substrate which had been produced post hydrolysis, a replicate from each sample type underwent AD treatment for a shorter period of time to try to avoid a build-up of these metabolites in the growth substrate (15 days after run 1 and 4 days for experimental run 2), and final samples were taken after 21 days for metabolomic analysis. Gas chromatography was performed to confirm the quantity of volatile fatty acids (VFAs) to gauge level and stage of breakdown by AD. Ideally it was hoped that the samples which had an initial pH of 6 would have a greater content of VFAs due to the inhibition of methanogens. A Hewlett Packard 5890 Series 2 GC with a Supelco capillary column was used. The column length was 30 m, with an internal diameter of 0.25 mm and a fused silica film of 0.25 μm thickness. The carrier gas was hydrogen with a flow rate of 1 ml min^-1^. The injector temperature was 230°C with an approximate 10:1 split injection. The column temperature started at 100°C and increased by 10°C per minute until 225°C was reached; this was then held for 2 minutes giving a final run time of 14.5 minutes. The GC used a flame ionisation detector; the temperature of the detector was set at 300°C. VFA standards were run prior to the samples to gauge elution times for the identification of peaks in the samples. This data was used to determine the total VFAs in the sample.

### Pilot insect feeding trials

To gain preliminary results on the suitability of digested EFB as a substrate and test for toxicity, *Hermetia illucens* (black soldier fly; BSF) larvae were fed on digested EFB. One hundred newly emerged larvae (<24 h post eclosion) were added to 25 g of feed in clear plastic deli pots (12 oz; base 9.5 cm diameter) covered with a layer of muslin, which was secured by a plastic lid. Five holes approximately 3 mm diameter were punched through the plastic lid to provide ventilation. Larvae were monitored and feed was added as required by the larvae based on the amount and appearance of the feed remaining in the pot and the mass of the larvae. Larvae were maintained at 27°C, 70% r.h. throughout the trial.

After 17 days subsisting only on digested EFB, ten black soldier fly larvae were chosen randomly and their mean mass calculated. Feeding trials were carried out with digested EFB removed from AD experiments at day 15 (run 1), day 4 (run 2) and day 21 (run 2).

## Results

### Metabolomic analysis of pre-processed EFB

#### LC-HRMS

**Fig 2:**
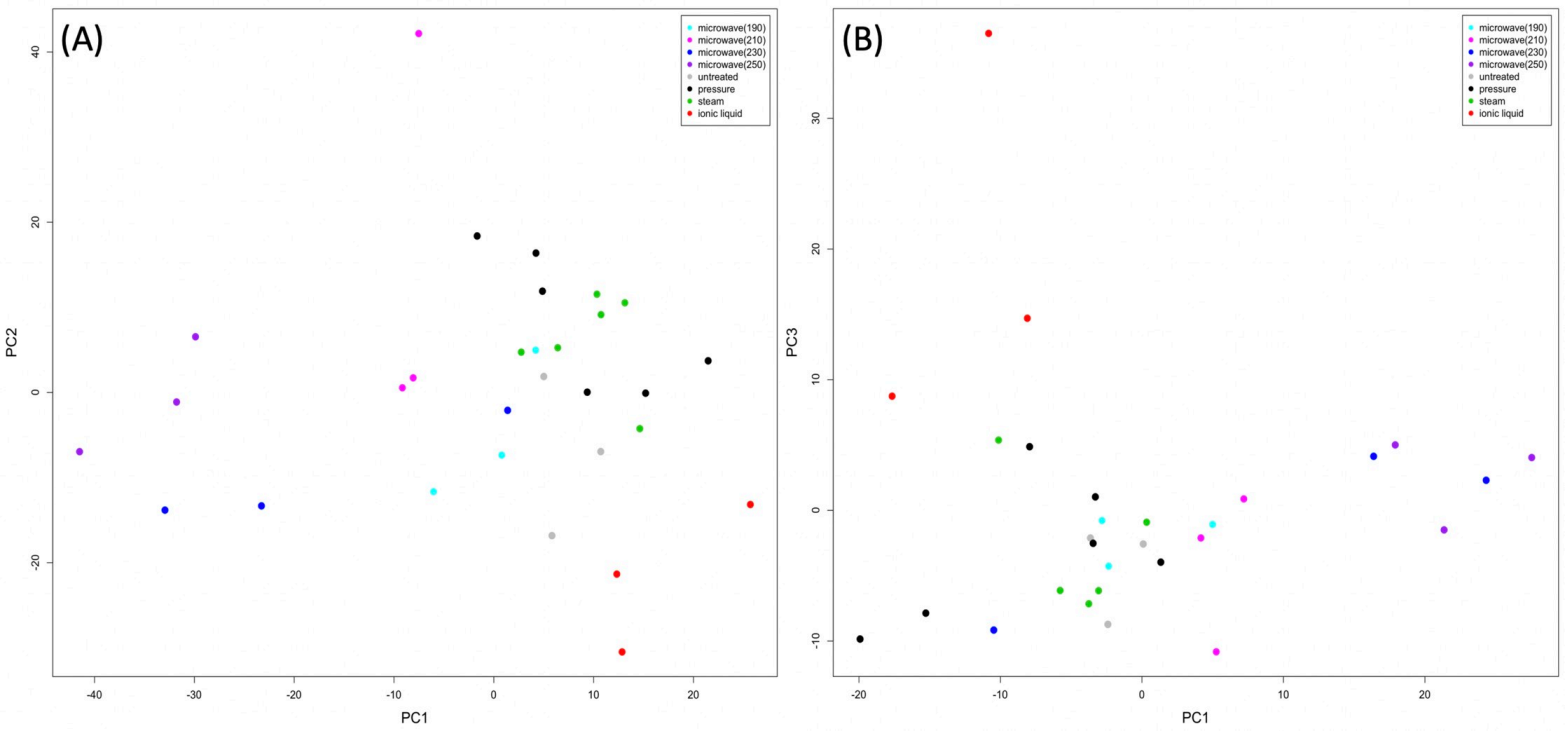
**PCA scores plots showing (A) positive mode and (B) negative mode LC–HRMS data from the pre–processed EFB**. One outlier, pre–processed by steam, was removed from the negative mode data. The first two principal components are shown for the positive data, whereas the first and third principal components are shown for negative data as the variance along the second component was due to a single (pressure–cooked) observation.

PCA of the LC-HRMS data in both positive and negative mode shows compositional differences between the pre-processing methods (Fig 2). The greatest variance (PC1) in both modes is due to differences between high temperature microwaved samples and other pre-processed/control samples. The loadings for PC1 showed the peaks contributing most are predominantly from species responsible for fruit flavours and fragrances, including metabolites found in cooked/roasted fruits, such as vanillin and derivatives and syringaldehyde, with higher intensities for samples microwaved at 250 and 230°C. In positive mode, peaks identified (confirmed with analytical standards) as the sugar levoglucosan, and fusaric acid, a mycotoxin from the fungus *Fusarium*, were also found to have higher intensities in samples microwaved at 250 and 230°C. PC2 shows separation of samples processed using ionic liquid (IL), mainly due to higher intensities for disaccharides in EFB samples pre-processed with IL.

For negative mode data, after removal of one outlier (steam), the variance along PC2 is still mainly due to differences in one pressure-cooked sample (not shown), but PC3 shows the same separation of samples processed using ionic liquid as seen along PC2 in positive mode data (Fig 2). Metabolites contributing most to the variance between pre-processing methods are shown in Table 4 together with their level of identification [22], with (unscaled) intensities plotted in S3 Fig and S4 Fig.

#### NMR

**Fig 3:**
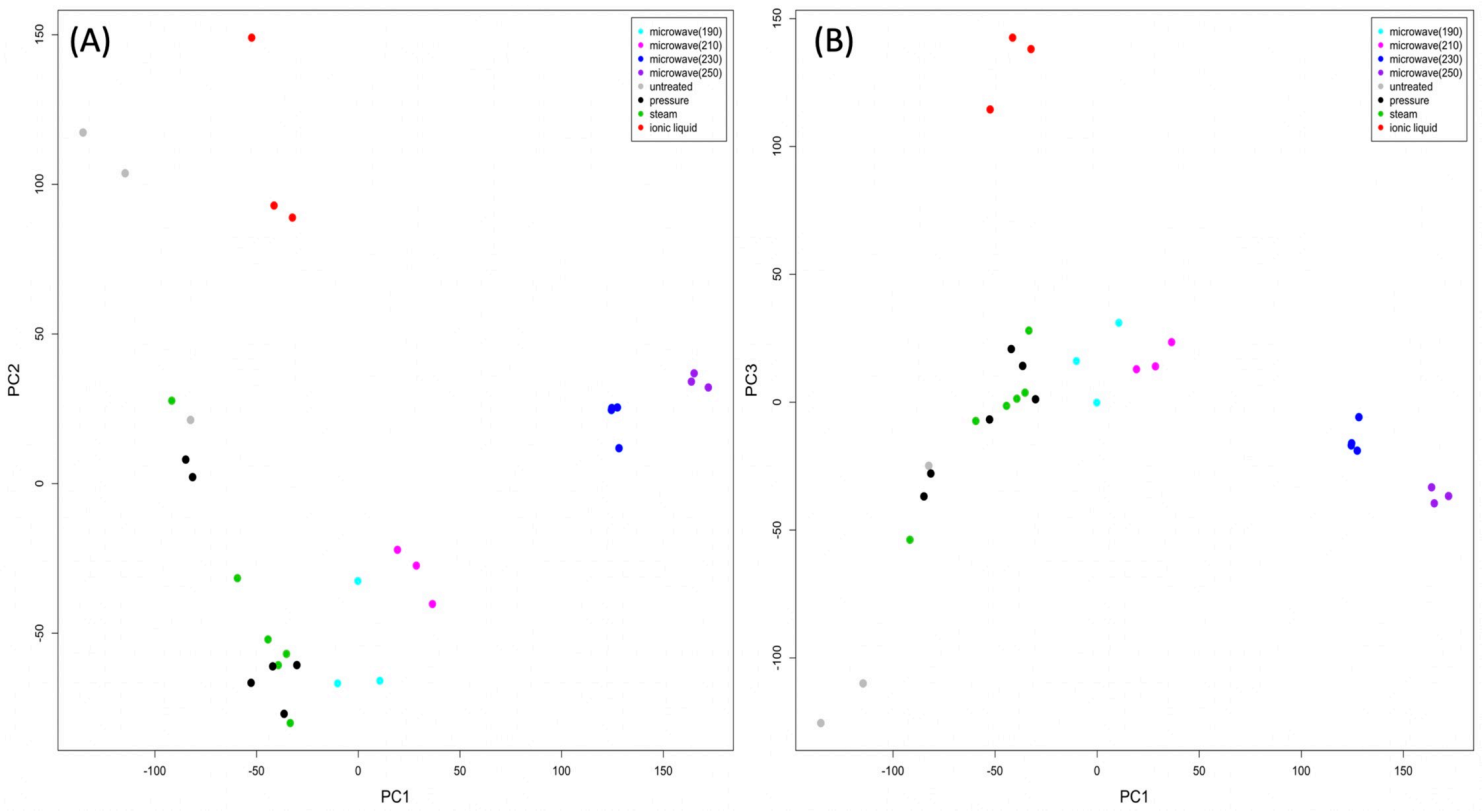
PCA scores plots showing 1D ^1^H–NMR data obtained from the pre–processed EFB. (A) shows the first two principal components and (B) shows the first and third principal components.

PCA scores plots of the ^1^H-NMR data show differences in composition between pre-processing methods (Fig 3). As with the LC-HRMS data, the greatest variance is due to differences in the EFB samples that were microwaved at 230°C and above (PC1). Regions related to this variance were matched to a variety of “Lignin and Cell Wall” compounds using the Biological Magnetic Resonance Data Bank [28], as well as fragrances and flavours, including vanillin (identified in LC-HRMS data and confirmed by analytical standard, Table 4). The concentrations of these compounds are higher in high temperature microwaved samples than for other pre-processing methods (S5 Fig), corroborating LC-HRMS results. PC2 shows some separation of IL samples, with highest concentrations of some compounds, most likely trilignols and tetralignols, in samples processed by moderate cooking methods (S6 Fig). This can be attributed to improved breakdown of lignin into dimers or monomers by high temperature microwaving or lignin removal by IL. The NMR spectrum obtained after processing with ionic liquids (S7 Fig) shows the absence of broader resonances from higher molecular weight components seen for other pre-processing methods. The separation of IL samples is clearer along PC3, from lower intensities for lignin compounds (tetralignins and trimers), and resonances from residual ionic liquid N,N dimethylbutylammonium hydrogen sulfate, confirmed by TOCSY to be present (S7 Fig, S8 Fig and S9 Fig).

Comparison of the HSQC spectra (S10 Fig) from EFB microwaved at 230°C and that of EFB pre-processed by ionic liquids shows overlapping peaks from lignin oligomers in the microwaved sample whereas simple sugars remain after lignin has been removed by IL.

### Anaerobic digestion

Although one month is a typical retention period for AD units, digestion of the EFB occurred faster than expected with most solids having been broken down after just two weeks. Due to this, the AD treatment was stopped after three weeks to prevent the substrate becoming too soupy for the larvae. End point samples (21 days) from the first AD run were not submitted for feeding trials as late digestion breakdown products, such as alcohols (Table 1), would be toxic to larvae and samples were removed earlier in the process for feeding trials in subsequent experiments. We found that samples digested at pH6 had greater quantities of volatile fatty acids (S3 Table), an indication that methanogens had been inhibited. The highest biogas production (per gram of VS used) was found for EFB pre-processed by IL and ran with a starting pH of 6, closely followed by microwaved (where only samples microwaved at 230°C were submitted for AD), both with a 2:1 digestate:EFB volatile solids (VS) ratio (S11 Fig). The lowest production of biogas was found for composted EFB, closely followed by milled EFB, steamed EFB (all with a 2:1 digestate:EFB VS ratio) and then microwaved EFB with a 1:2 digestate:EFB VS ratio.

### Metabolomic analysis of digested EFB

#### NMR

**Fig 4:**
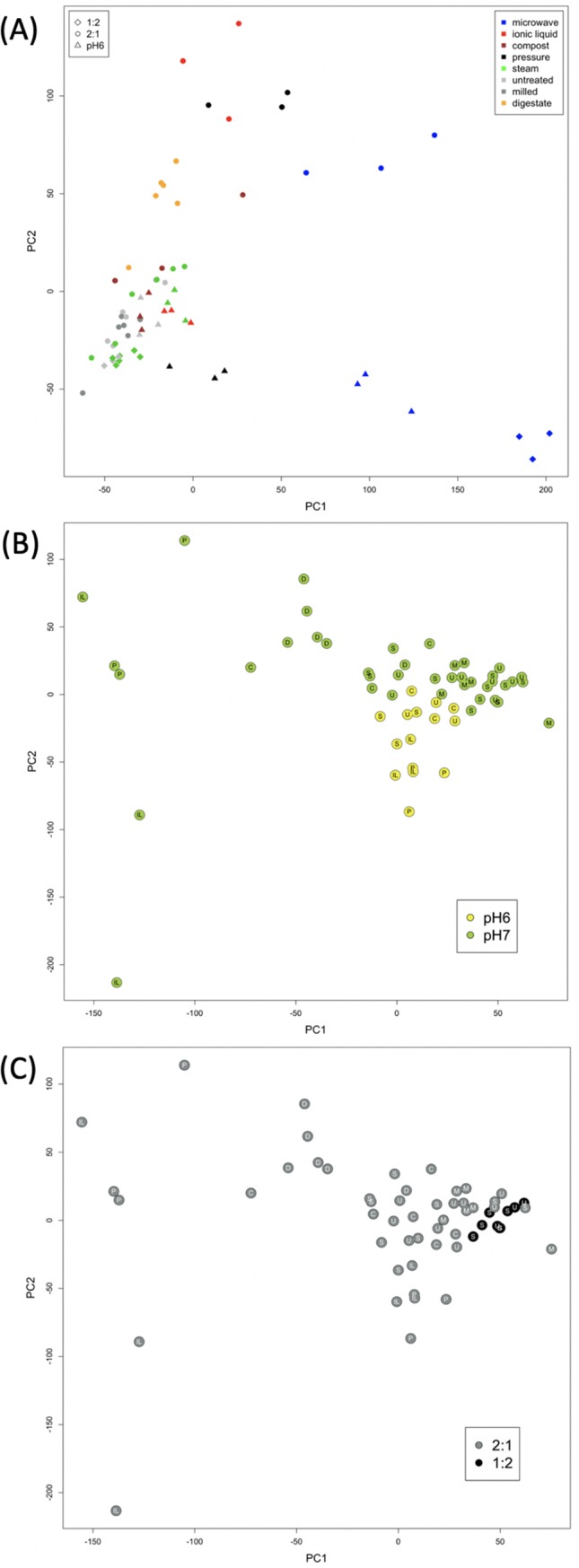
PCA scores plots showing 1D ^1^H–NMR data from the digested EFB. (A) shows the separation of microwaved samples, which have been removed before PCA in (B), coloured by pH, and (C) coloured by digestate:EFB ratio.

PCA on NMR data from digested EFB also showed separation of the microwaved samples (Fig 4) due to higher intensities for peaks corresponding to lignin dimers (S12 Fig) and lower peaks, attributed to fragrant compounds, such as eucalyptol and fenchol (S13 Fig). Due to the overlap of many resonances from high molecular weight lignin and its various polymer subunits, confirmation of the specific metabolites causing the distinction between groups of samples was not performed by NMR spectroscopy using analytical standards. Instead, resonances were tentatively assigned using database searches and 2D NMR spectra. After removing microwaved samples, PCA revealed clustering of observations according to pH with samples processed by pressure cooking or using ionic liquid showing the greatest differences due to the acidity of digestion (Fig 4). Metabolites potentially responsible for the differences include lignin dimers and monomers, higher at pH6, as well as xylose, a derivative of vanillin (both identities confirmed in LC-HRMS) and eugenol, lower at pH6 (S14 Fig). The microwaved sample digested at pH6 shows particularly high intensity for a large peak potentially corresponding to a lignin dimer (S15 Fig). The composted and control groups, that were also digested at both pH6 and pH7, show little difference due to pH level.

**Fig 5:**
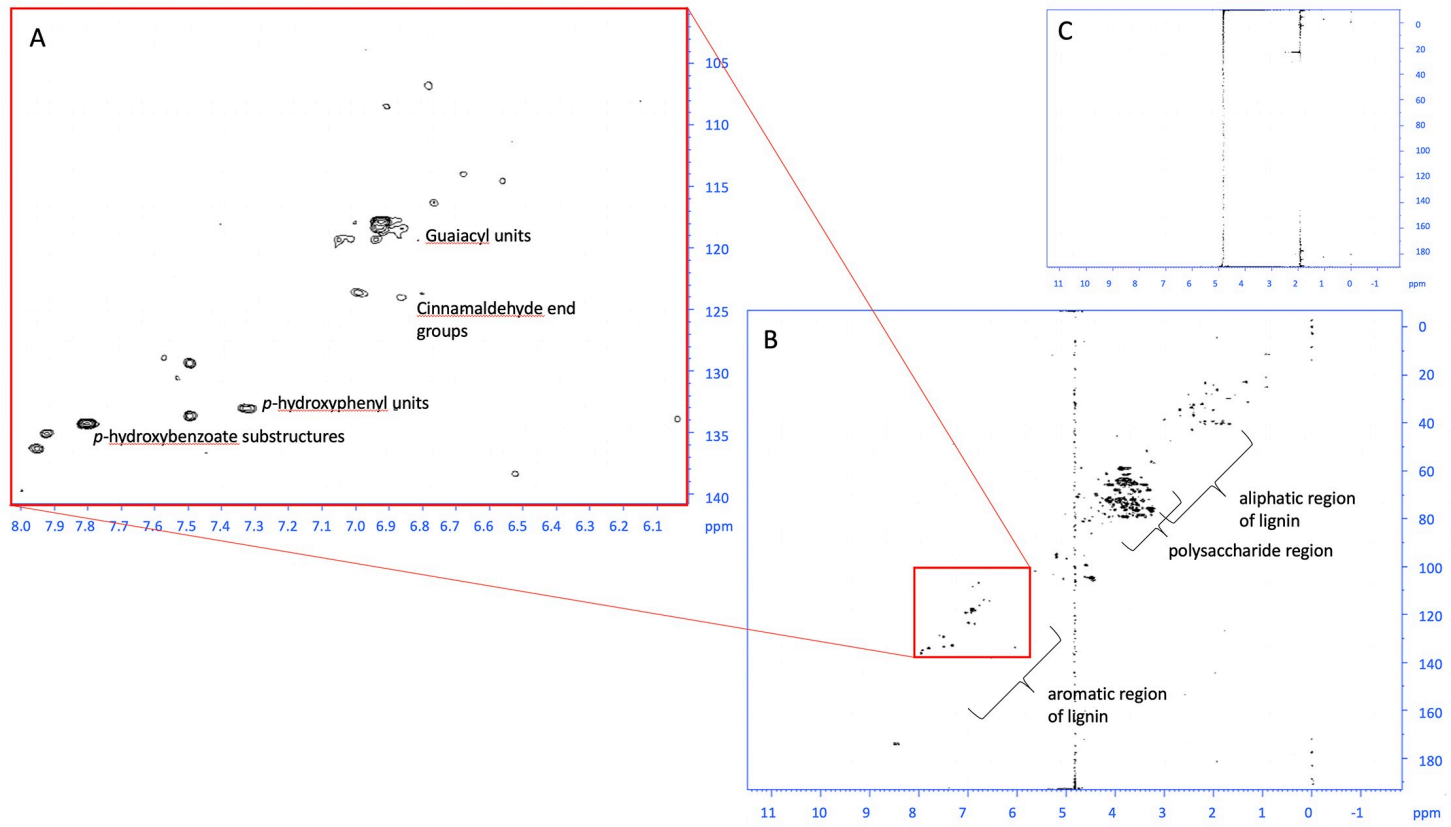
**HSQC spectra from EFB sample pre–processed by microwaving at 230°C before (A and B) and after digestion (C)**. Expansion of the aromatic region is shown in A, showing common lignin polymer linkages, which are no longer observed after AD (C).

Although with much lower intensity, the TOCSY of the digested EFB pre-processed by IL shows the same spin system present before AD, showing that traces of IL are still present after AD (S16 Fig). Comparison of HSQC spectra before and after AD for the samples pre-processed by microwaving shows diagnostic peaks for lignin unit linkages [29] and ends of oligomers in the aromatic region before AD, which are not seen in the spectrum after digestion, providing evidence of greater breakdown of lignin oligomers by AD (Fig 5).

#### LC-HRMS

No consistent compositional differences between pre-processing methods were apparent after AD (S17 Fig and S18 Fig). As LC-HRMS experiment here measures the small molecular weight constituents, the lack of differences between samples supports the fact that differences in NMR were due to high molecular weight compounds, such as lignin. The mycotoxin fusaric acid, identified in the pre-processed EFB, was not detected in the digested EFB LC-HRMS data.

### Modelling biogas production

**Fig 6:**
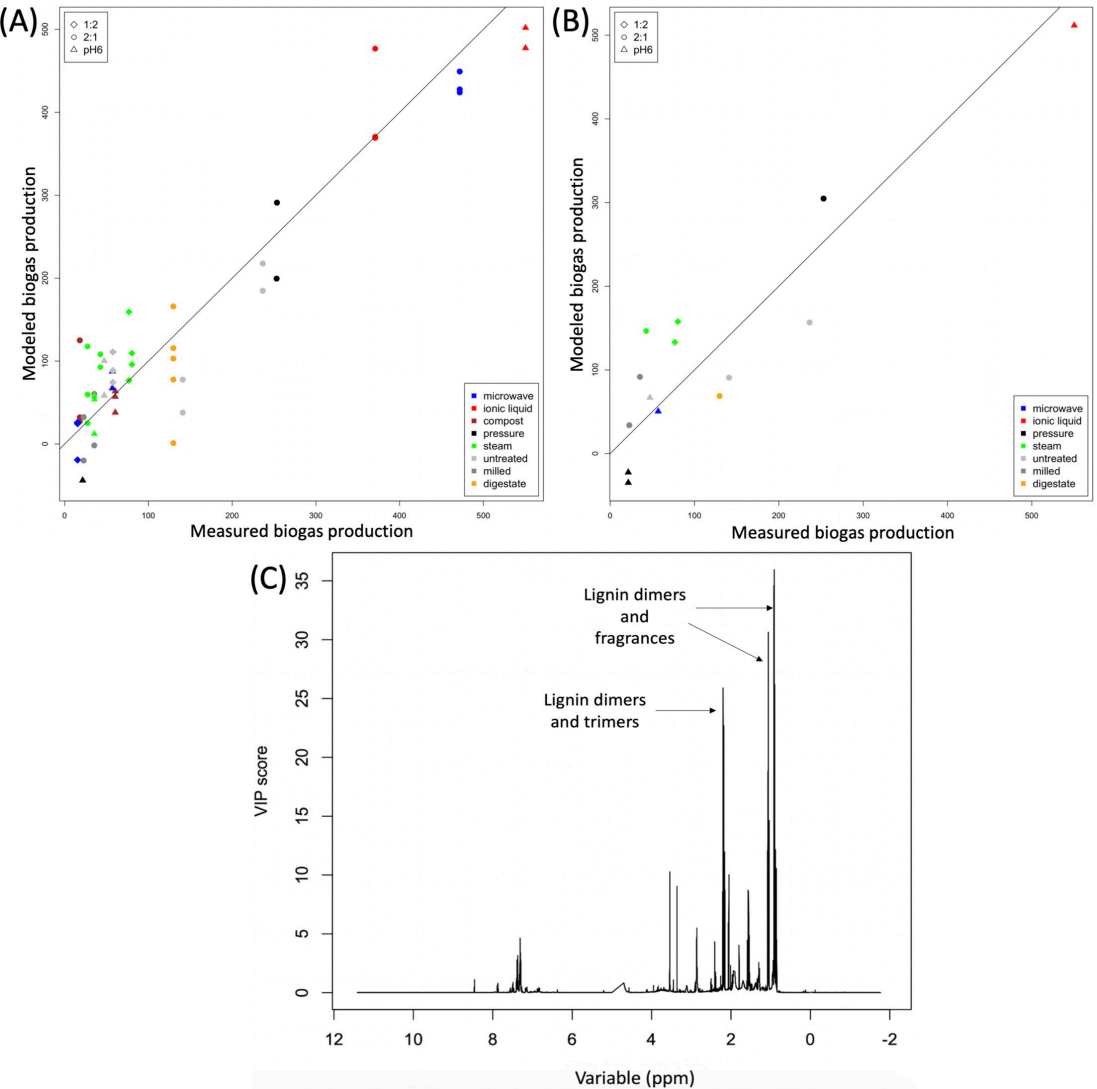
Results of PLS–R using 1D NMR data to model biogas production. A relationship is shown between peaks in the NMR spectra (after AD) and the amount of biogas produced for (A) the training data and (B) an independent test set. Variable importance in projection (VIP) scores are shown in (C). Large VIP scores (VIP score> 25) on the plot indicate the specific regions or peaks of the NMR spectrum that are related most to how much biogas yield.

Partial least squares regression (PLS-R) was performed to relate peaks in the NMR spectra (obtained after digestion) to biogas production (Fig 6). Cross-validation was repeated ten times, with 75% of the data randomly assigned as training data each time and the remaining 25% used as test data. For the test data, we obtained a mean R^2^ value of 0.63 (RMSEP = 92.96), which when compared to the fit for the training data, R^2^ = 0.65 (RMSEP = 84.41), shows the model is not overfitting. The modelled biogas production is plotted against the measured biogas in Fig 6. It can be seen that pre-processing with ionic liquids or using a 2:1 digestate to EFB ratio after microwaving produces most biogas, i.e. samples with a low concentration of lignin polymers after AD. On the other hand, microwaved samples undergoing AD at pH6 or at a 1:2 ratio produce little biogas in comparison, as do other samples in these conditions. The variable importance in projection (VIP) scores in Fig 6C show the specific regions of the NMR spectrum, those with the largest VIP scores, that are related to biogas output. These regions or peaks with VIP score >25 were found to have the same chemical shifts as those identified in PCA, most likely to be assigned to lignin dimers and trimers.

### Preliminary insect feeding trials

A heating fault in the insectory led to a significant temperature drop in the first feeding trial, involving digested EFB taken at day 15, causing larvae to die before any results could be noted. For the second AD run, samples were removed for feeding trials at day 4 and larvae survived on digested EFB from all pre-processing methods. Although growth was minimal over the 17 day period in comparison to control larvae fed on chicken feed (usual substrate for larvae used to maintain the colony), it is likely that toxic compounds are not present in the digested EFB at this stage. In terms of growth, the best results were obtained for larvae fed on EFB pre-processed by IL (mean mass of 10 larvae = 130 mg), particularly when digested at pH6, whereas composted EFB gave the worst results (mean mass of 10 larvae = 35 mg, S19 Fig). For comparison, the mass of ten larvae on chicken feed after 14–18 days would be approximately 2000 mg.

For samples taken at day 21, larvae did not survive when fed on four of the digested EFB substrates (digestate control only (no EFB), milled, composted and ionic liquids) and, for larvae that did survive, often fewer than ten were large enough to measure growth.

The only substrate digested for 21 days at pH7 on which larvae survived was EFB that was not pre-processed. As few larvae survived, only 5 contributed to each replicate measurement although the masses obtained were similar to those for untreated EFB digested for just 4 days. All other substrates on which larvae survived were digested at pH6 with untreated EFB being the only sample digested for 21 days giving better results than obtained for 4-day digestion. Composted samples, digested at pH 6, gave similar larval mass when digested for 4 or 21 days, but larvae fed on EFB pre-processed by ionic liquid, digested at pH6, for 21 days were much lower in mass than larvae fed on the same samples after just 4 days digestion.

## Discussion

Distinctive aromas were particularly noticeable during pre-processing experiments that involved heating EFB and therefore the identification of metabolites responsible for flavours and fragrances is not unexpected. The higher concentrations of vanillin and syringaldehyde detected in microwaved samples can be explained as the thermal depolymerization of lignin [30–33], both being indicators of burning biomass [34]. It follows that increased temperatures and pressures increase the breakdown of lignin in EFB.

Another compound associated with the pyrolysis of lignocellulosic biomass, levoglucosan, is only detected in the high temperature (≥230°C) microwaved EFB samples. It has been shown that Maillard reaction products such as levoglucosan are toxic to some microorganisms, reducing the growth of many bacterial colonies [35]. This will in turn change the microbial population during AD and could explain the lower biogas production for EFB microwaved at 230°C after digestion in the ratio 1:2 digestate:EFB. With a higher proportion of EFB present during AD, it could be that colony growth, including microorganisms producing biogas, has been inhibited by the presence of bacteriocidal levoglucosan,

Malic acid, shown to be important in producing fatty acids in palm fruit [36], was found to increase with pre-processing temperature, being highest for microwaved samples in line with the use of microwave-assisted hydrothermal (MH) techniques for the extraction of malic acids from plants and fruit wastes [37].

Sugar content was found to vary between pre-treatment methods, with higher concentrations of glucose and xylose detected in high temperature microwaved EFB, indicating greater breakdown of cellulose, hemicellulose and other disaccharides and polysaccharides. Glucose was confirmed by analytical standards (in LC-HRMS) and is clearly evident in the 1D ^1^H NMR spectrum of microwaved samples. Rather than converting sugars into monomers and simultaneous Maillard reaction products via application of heat, processing with ionic liquid removes the lignin to leave cellulose, a polymer of *D*-glucose subunits. We found the highest concentration of disaccharides after pre-treatment by IL, which after hydrolysis during AD resulted in the best proliferation of microbial communities and gave the most promising results in larvae feeding trials. The higher concentration of disaccharides in EFB pre-processed by IL is most likely to be from the degradation of polymeric hemicellulose that was not removed by the IL and remained after pre-processing, and from amorphous cellulose. The presence of trace IL detected by 1D and 2D NMR spectroscopy was not detrimental to the bacterial communities during AD, nor did it have a negative effect on larval growth. As the environmental risk and toxicity of ionic liquids has been raised [38], it is encouraging to see that the ionic liquid used here is not toxic to the larvae.

It could be that the higher levels of the mycotoxin fusaric acid observed in microwaved samples are due to increased extraction as a result of greater sample breakdown. As the waste biomass, left unmonitored by the palm oil industry, can be infected with *Fusarium*, the presence of fusaric acid in EFB is not surprising, but that this mycotoxin could subsequently enter the food chain is of concern. It is therefore encouraging that this mycotoxin is not detected after anaerobic digestion. Although the EFB is less concentrated, in some AD experiments in the ratio of 1:2 with the straw inoculant, fusaric acid should still be detected by LC-HRMS if present.

Due to the small-scale preliminary feeding trials, small size of the larvae and the semi-liquid nature of the digested EFB it was very difficult to assess an accurate survival rate, however, preliminary larvae feeding trials using EFB digested for 4 days suggested that this substrate was unlikely to be toxic. Although further investigation would be necessary to verify the absence of mycotoxins in the larvae themselves after feeding on waste biomass, the inevitable fungal infection of waste biomass did not produce a toxic substrate for the larvae. Preliminary feeding trial results also suggested that end of digestion products such as alcohols could be toxic to larvae. Larvae did not survive on half of the EFB samples digested for 21 days, and conditions in which they did survive had few surviving larvae. The results provide some evidence that digestion at pH6 does inhibit methanogenic microorganisms and favour hydrolytic, acidogenic and acetogenic microorganisms. Interestingly, the highest mean larval mass was obtained for untreated EFB digested for 21 days at pH6, although the standard error is high. It is possible that the lack of pre-processing led to slower digestion, therefore less toxic end of digestion products had been generated than in other samples.

These conditions produced very little biogas, whereas the EFB pre-processed by IL and digested for 4 days at pH6 not only showed promise in preliminary feeding trials, also produced most biogas, peaking in biogas production around day five (S11 Fig). The results of the PLS-R analysis suggest that biogas production is related to lignin content. Pre-processing methods that either remove lignin (IL) or break down the lignin polymers into much smaller units (microwaving) led to greatest biogas production (Fig 6). VIP scores confirm the relationship between biogas production and regions of the spectrum related to lignin dimers and trimers as well as sugars.

In conclusion, we have identified optimal conditions under which anaerobic digestion of EFB has the potential to provide substrate for farmed insects in addition to producing biogas. The use of complementary analytical techniques allowed the breakdown of this waste biomass to be explored. LC-HRMS allowed compositional differences between pre-processing methods to be identified prior to AD, such as increased levels of bacteriocidal levoglucosan. By measuring biogas production, we were able to observe the effect of this compound on the microbial community. Our NMR results after AD show the main differences between pre-processing methods are related to the concentration of lignin dimers, trimers and tetralignols. This is corroborated by the fact that no consistent differences between pre-processing methods after AD were identified by LC-HRMS, as such high molecular weight compounds would not be detected by this technique. The use of 2D NMR spectroscopy provided further evidence of increased lignin breakdown after pre-processing by microwaving and IL and aided identification of remaining IL, which preliminary survival trials showed is not toxic to larvae. Comparison with HSQC spectra in the literature [29, 39, 40] allowed diagnostic lignin unit linkage peaks within the aromatic region to be identified. For microwaved samples, the presence of these peaks before AD and absence after AD (Fig 5) shows that the lignin has been successfully broken down into smaller units such as dimers and monomers. However, microwaving is not optimal in terms of the effect on microbial communities as well as being expensive. Preference was for lower energy and cost/recyclable processes, such as steaming or heating the EFB under pressure, as these pre-processing methods would be transferrable to oil palm mills in developing countries, which generate heat and steam already as part of their industrial processes [4]. To conclude, we found that pre-processing EFB using ionic liquid before anaerobic digestion at pH6 with a 2:1 digestate:EFB ratio for 4–5 days could provide a nutritious substrate for insect farming whilst simultaneously producing more biogas than other conditions. Moreover, the method is both economically viable and environmentally friendly, particularly as the ionic liquid can be recovered and recycled [18], which could improve the mass-energy balance of any oil palm mills, should these practices be adopted in industry. Further work, involving the scaling up the digestion of EFB, perhaps mixed with other biomass, is required to allow full feeding trials to take place.

## Supporting information

S1 Fig **PCA scores plots obtained from LC-HRMS negative mode (A and B) and positive mode (C and D) scaled data from pre-processed EFB (before AD), with each sample coloured by batch and QC samples indicated by squares**. A and C show that batch differences dominate the variance. After batch correction techniques have been applied, B and D show no distinction between batches.
(TIF)

S2 Fig **PCA scores plots obtained from LC-HRMS negative mode (A and B) and positive mode (C and D) scaled data from digested EFB, with each sample coloured by batch.** A and C show the greatest source of variance is the separation of the QC samples (indicated by squares), with differences between batches along PC2. After batch correction techniques have been applied, B and D show no distinction between batches.
(TIF)

S3 Fig Peak intensities (unscaled) for compounds identified by LC-HRMS of pre-processed EFB (before AD). The m/z and retention time (in minutes) is given as “X *m/z*_r*etentiontime*”. Small negative values for some observations are due to batch correction.
(TIF)

S4 Fig Peak intensities (unscaled) for compounds identified by LC-HRMS of pre-processed EFB (before AD). The m/z and retention time (in minutes) is given as “X *m/z*_r*etentiontime*”. Small negative values for some observations are due to batch correction.
(TIF)

S5 Fig Examples of peaks with highest intensities (unscaled) in microwaved samples, identified in NMR data from pre-processed EFB (before AD). Chemical shifts (shown above plots) were matched to a variety of lignin and cell wall compounds as well as fragrances and flavours, using the Biological Magnetic Resonance Data Bank [28].
(TIF)

S6 Fig Examples of peaks with lowest intensities (unscaled) in samples processed using ionic liquids, identified in NMR data from pre-processed EFB (before AD). Chemical shifts are shown above plots. The most likely species are trilignols and tetralignols.
(TIF)

S7 Fig ^**1**^**H-NMR spectra obtained from EFB pre-processed by ionic liquids (red) and by microwaving at 230**°**C (blue).** The five resonances assigned to the ionic liquid are indicated on the spectrum, with the protons responsible (1–5) shown on the structure of the ionic liquid N,N dimethylbutylammonium hydrogen sulfate (top left).
(TIF)

S8 Fig Examples of peaks with highest intensities (unscaled) in samples processed using ionic liquids, identified in NMR data from pre-processed EFB (before AD). Chemical shifts (shown above plots) correspond to the structure of the ionic liquid N,N dimethylbutylammonium hydrogen sulfate.
(TIF)

S9 Fig 2D NMR 1H-1H TOCSY obtained from EFB pre-processed by ionic liquids. The expanded region shows the spin system from the trace ionic liquid N,N dimethylbutylammonium hydrogen sulfate present, with assignments as shown in S5 Fig.
(TIF)

S10 Fig **HSCQ obtained for EFB pre-processed by microwave (A) and IL (B) before anaerobic digestion.** These show peaks for sugars and lignin oligomers in the microwaved sample, but not in that pre-processed by IL.
(TIF)

S11 Fig Mean cumulative biogas production for each AD run (1–3), reported in ml per gram of volatile solid, after subtraction of mean digestate value. In run 1 (A), an increase in biogas production is seen at day 15, when one sample was removed for feeding trials, suggesting that this sample was producing biogas at a slower rate than the remaining two. Key: M = microwave, P = Pressure, S = Steam, C = Compost, IL = Ionic Liquid, EFB = Untreated EFB (no pre-processing), MI = Milled. Where digestate:EFB ratios are given (2:1 or 1:2), AD was carried out at pH7; where pH6 is shown, the ratio is 2:1.
(TIF)

S12 Fig Examples of peaks with highest intensities (unscaled) in microwaved samples, identified in NMR data from digested EFB (after AD). Chemical shifts (shown above plots) were attributed to lignin dimers.
(TIF)

S13 Fig Examples of peaks with lowest intensities (unscaled) in microwaved samples, identified in NMR data from digested EFB (after AD). Chemical shifts (shown above plots) were matched to fragrances/flavours, possibly eucalyptol and fenchol.
(TIF)

S14 Fig Examples of peaks showing differences in intensities (unscaled) due to pH, identified in NMR data from digested EFB (after AD). When pre-processed by ionic liquid, pressure cooking or microwaving, intensities for samples undergoing AD at pH6 are lower than for samples undergoing AD at pH7 (both at 2:1 digestate:EFB ratio).
(TIF)

S15 Fig Examples of peaks showing differences in intensities (unscaled) due to pH, identified in NMR data from digested EFB (after AD). For the peak at 0.9 ppm, when pre-processed by ionic liquid or pressure cooking, samples undergoing AD at pH6 are much lower than for samples undergoing AD at pH7 (both at 2:1 digestate:EFB ratio). For the peak at 3.36 ppm, intensities are higher for samples pre-processed by microwaving, pressure cooking or steaming and digested at pH6.
(TIF)

S16 Fig 2D NMR 1H-1H TOCSY obtained for EFB after pre-processing by ionic liquids and after anaerobic digestion. The ionic liquid, N,N-dimethylbutylammonium hydrogen sulfate, can still be seen after digestion.
(TIF)

S17 Fig PCA scores plots for the first two principal components obtained from positive mode LC-HRMS data from the EFB after AD, with observations coloured by pre-processing method. No consistent differences between pre-processing methods can be seen.
(TIF)

S18 Fig PCA scores plots for the first two principal components obtained from negative mode LC-HRMS data from the EFB after AD, with observations coloured by pre-processing method. No consistent differences between pre-processing methods can be seen.
(TIF)

S19 Fig **Larval mass for preliminary feeding trials at day 4 (A) and day 21 (B) of the second AD run, all 2:1 digestate:EFB ratio.** Unless indicated, the average mass of ten larvae is given for each of three replicate measurements. Key: D = digestate only, M = Milled EFB, C = Composted EFB, UM = Untreated Control (not milled) EFB, IL = EFB treated with IL. AD at pH7 except for C6, UM6 and IL6 at pH6. α = only five larvae included in each replicate measurement, β = only seven-nine larvae included in each replicate measurement.
(TIF)

S1 Table Mean Bhattacharrya distance between PCA scores for different LC-HRMS batches, before and after removal of batch differences. *one steamed sample was removed as an outlier from this analysis.
(PDF)

S2 Table Dosing quantities and experimental conditions for each AD experiment. (PDF)

S3 Table Total volatile fatty acid (VFA) of digested EFB samples. (PDF)
