# Supplementary figures and images for: From waste to food: Optimising the breakdown of oil palm waste to provide substrate for insects farmed as animal feed
Source: PLoS One. 2019 Nov 7;14(11):e0224771. doi: 10.1371/journal.pone.0224771 (PMC6837394; doi:10.1371/journal.pone.0224771)

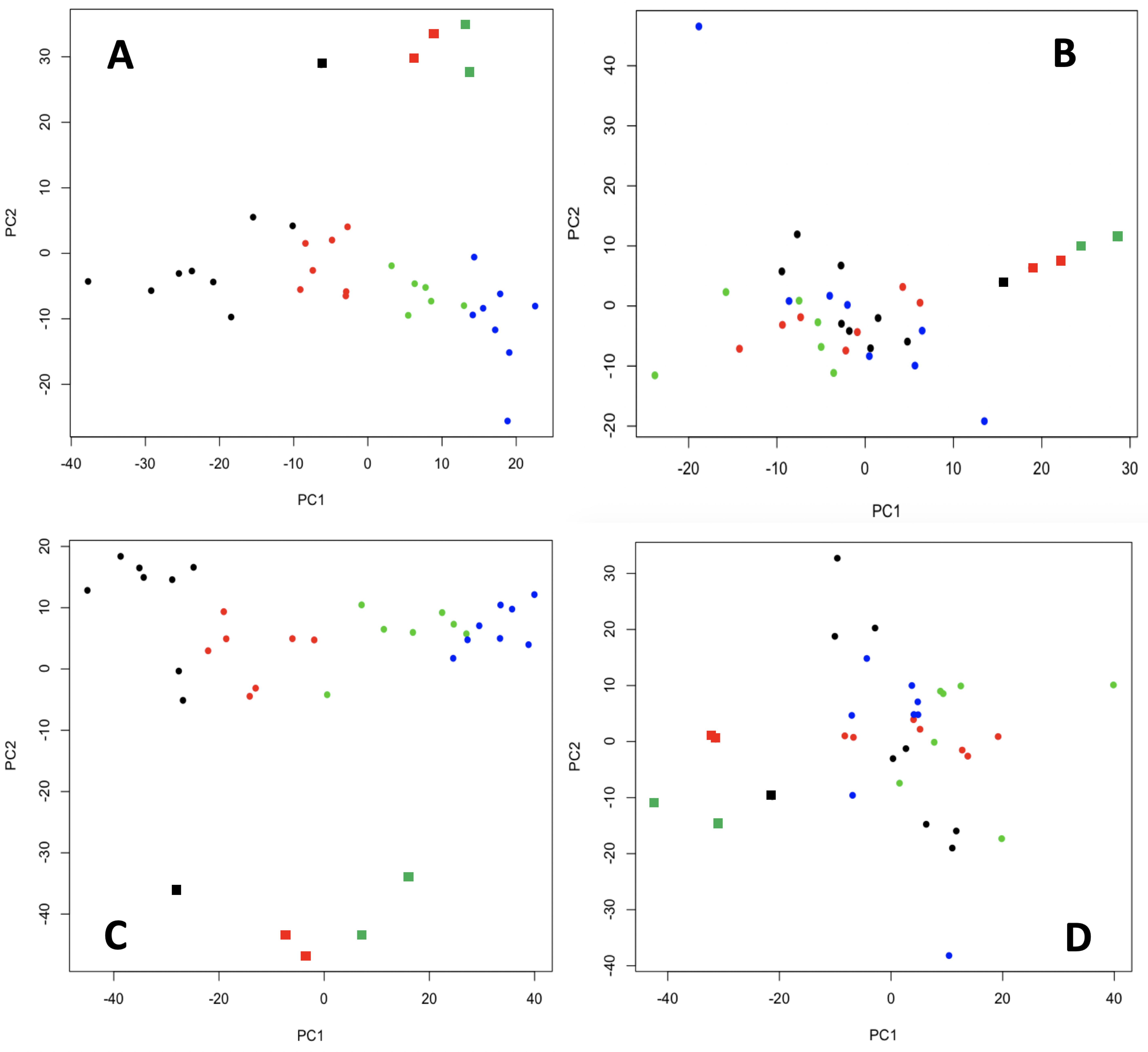

Supplement: S1 Fig — PCA scores plots obtained from LC-HRMS negative mode (A and B) and positive mode (C and D) scaled data from pre-processed EFB (before AD), with each sample coloured by batch and QC samples indicated by squares. A and C show that batch differences dominate the variance. After batch correction techniques have been applied, B and D show no distinction between batches. (TIF) [file pone.0224771.s001.tif]

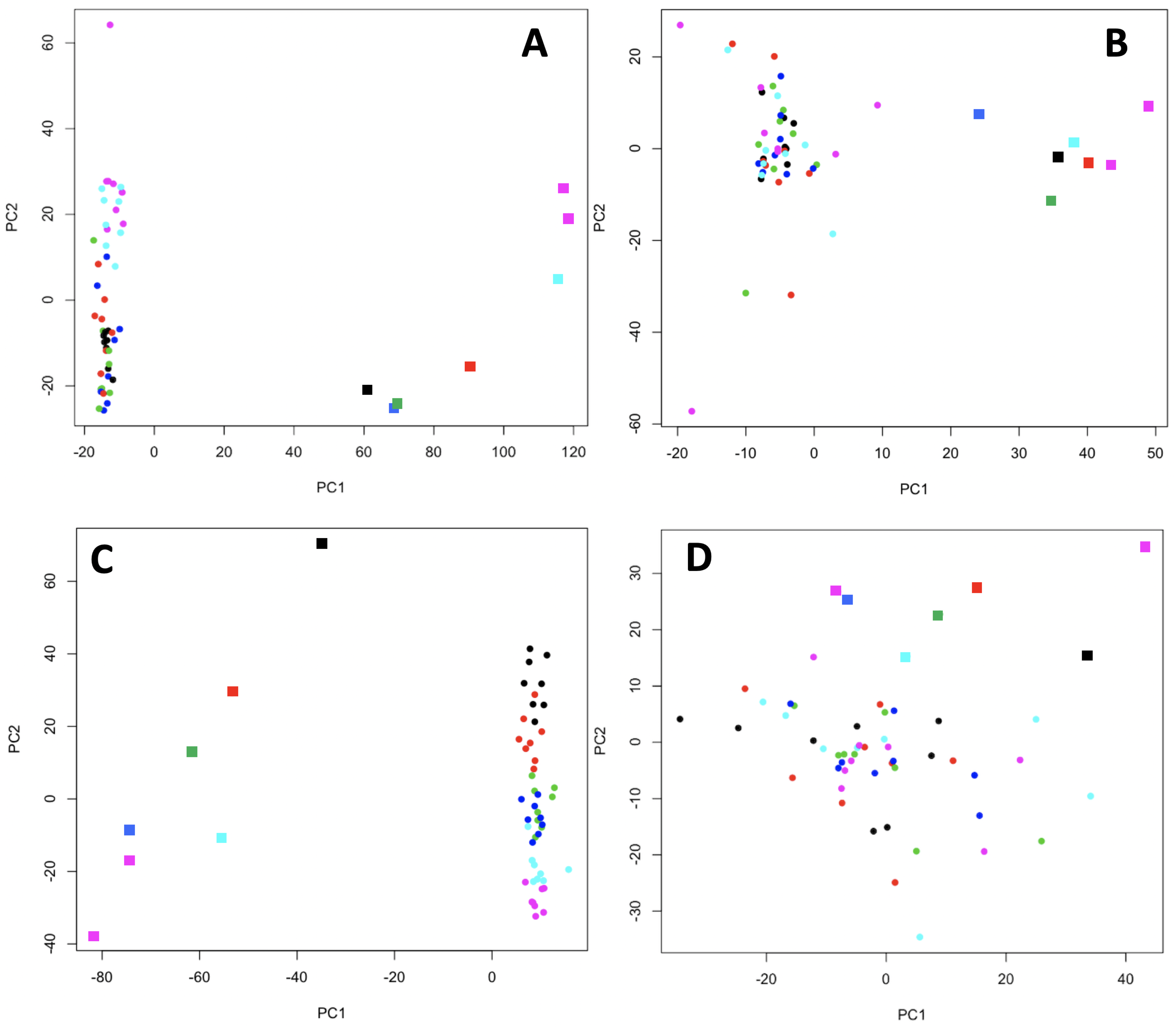

Supplement: S2 Fig — PCA scores plots obtained from LC-HRMS negative mode (A and B) and positive mode (C and D) scaled data from digested EFB, with each sample coloured by batch. A and C show the greatest source of variance is the separation of the QC samples (indicated by squares), with differences between batches along PC2. After batch correction techniques have been applied, B and D show no distinction between batches. (TIF) [file pone.0224771.s002.tif]

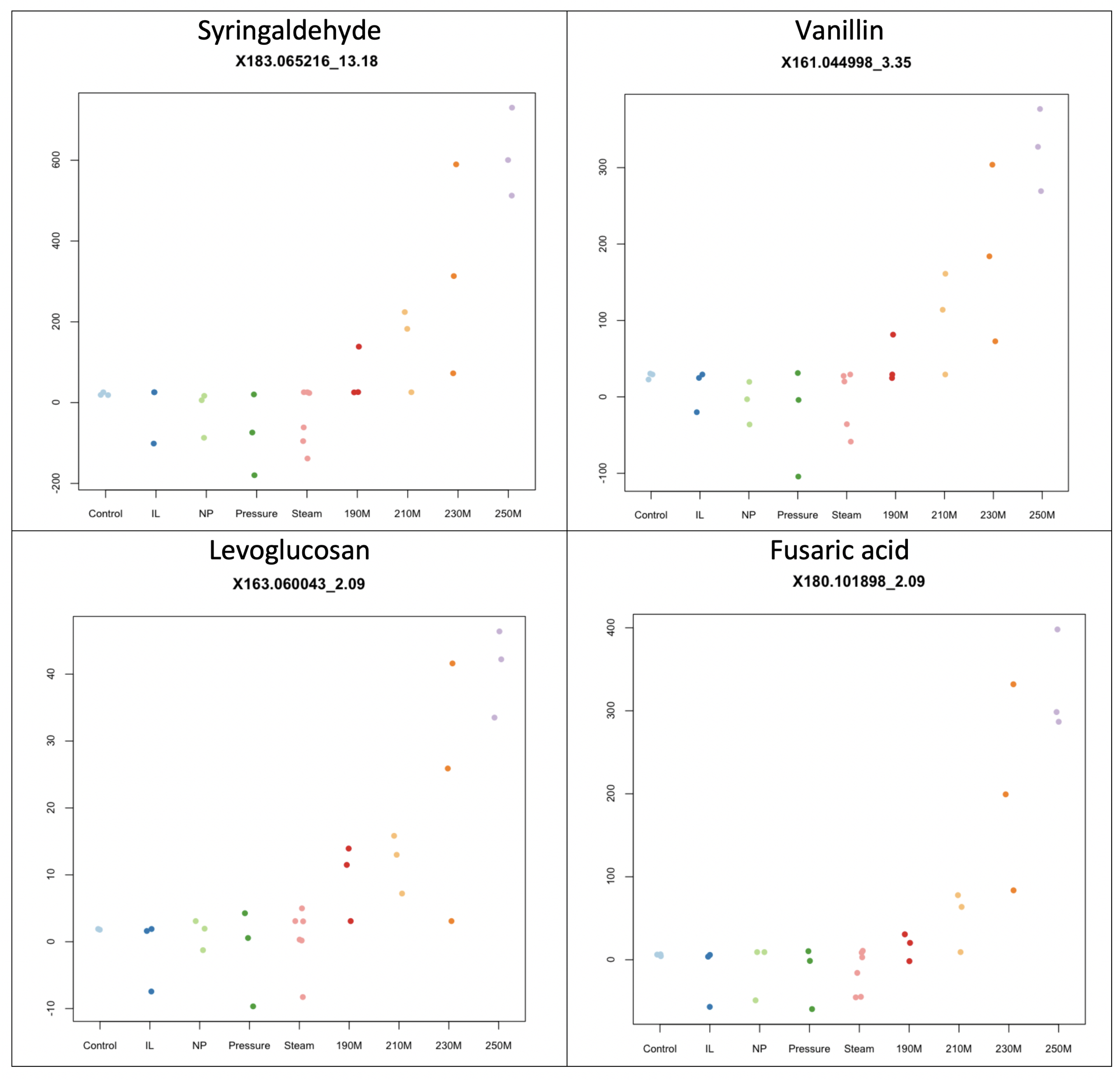

Supplement: S3 Fig — The m/z and retention time (in minutes) is given as “X m/z_retentiontime”. Small negative values for some observations are due to batch correction. (TIF) [file pone.0224771.s003.tif]

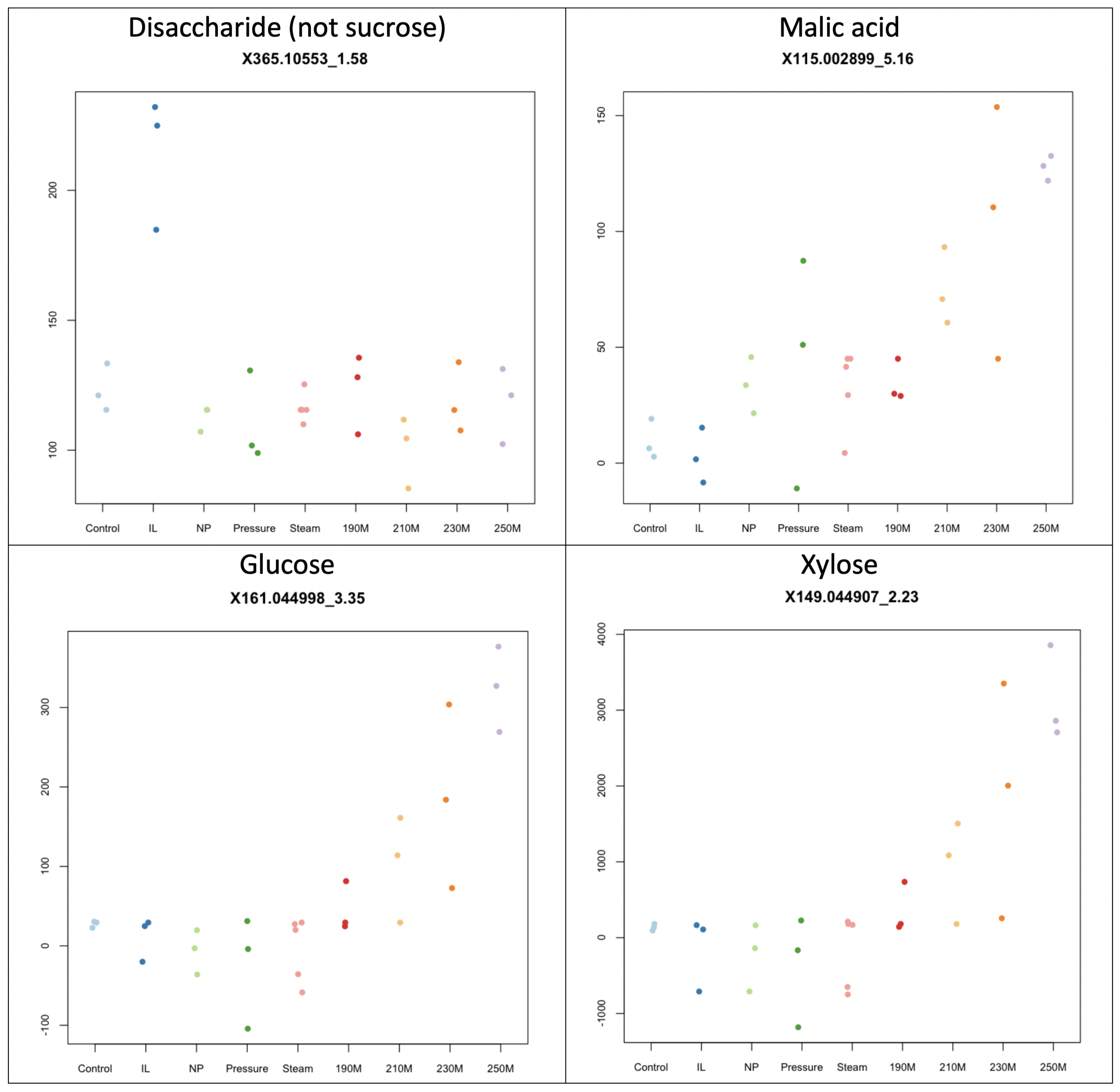

Supplement: S4 Fig — The m/z and retention time (in minutes) is given as “X m/z_retentiontime”. Small negative values for some observations are due to batch correction. (TIF) [file pone.0224771.s004.tif]

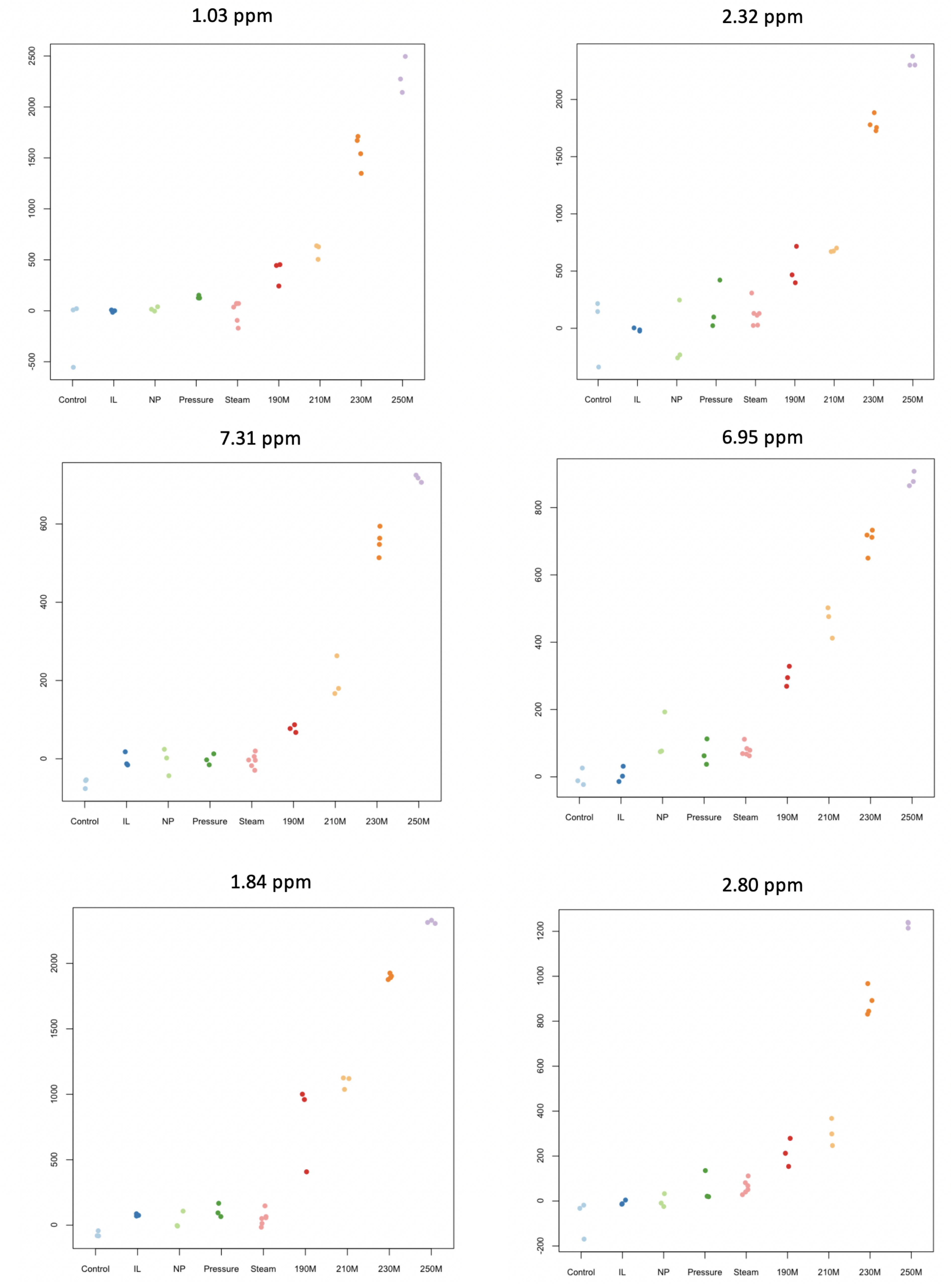

Supplement: S5 Fig — Chemical shifts (shown above plots) were matched to a variety of lignin and cell wall compounds as well as fragrances and flavours, using the Biological Magnetic Resonance Data Bank [28]. (TIF) [file pone.0224771.s005.tif]

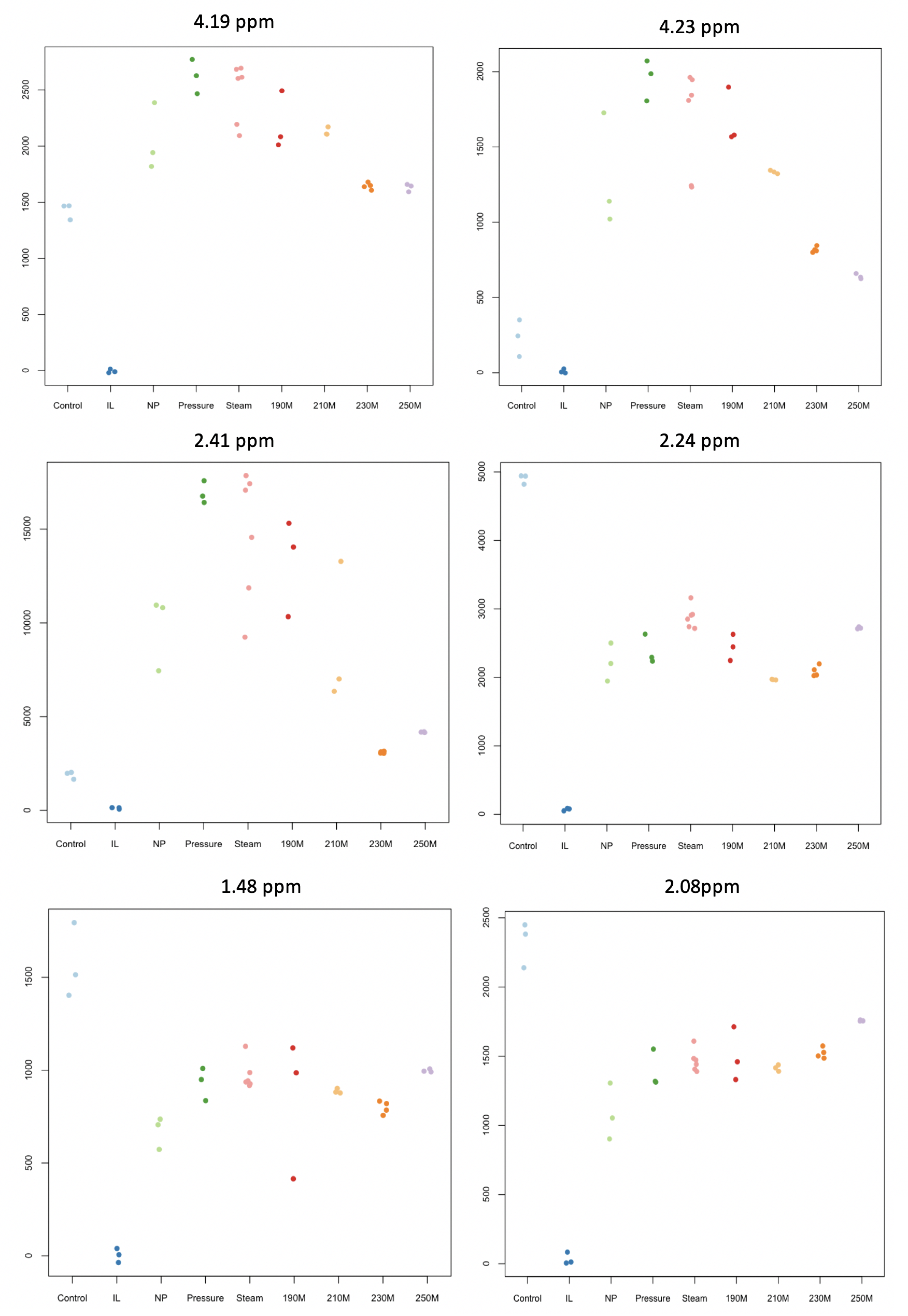

Supplement: S6 Fig — Chemical shifts are shown above plots. The most likely species are trilignols and tetralignols. (TIF) [file pone.0224771.s006.tif]

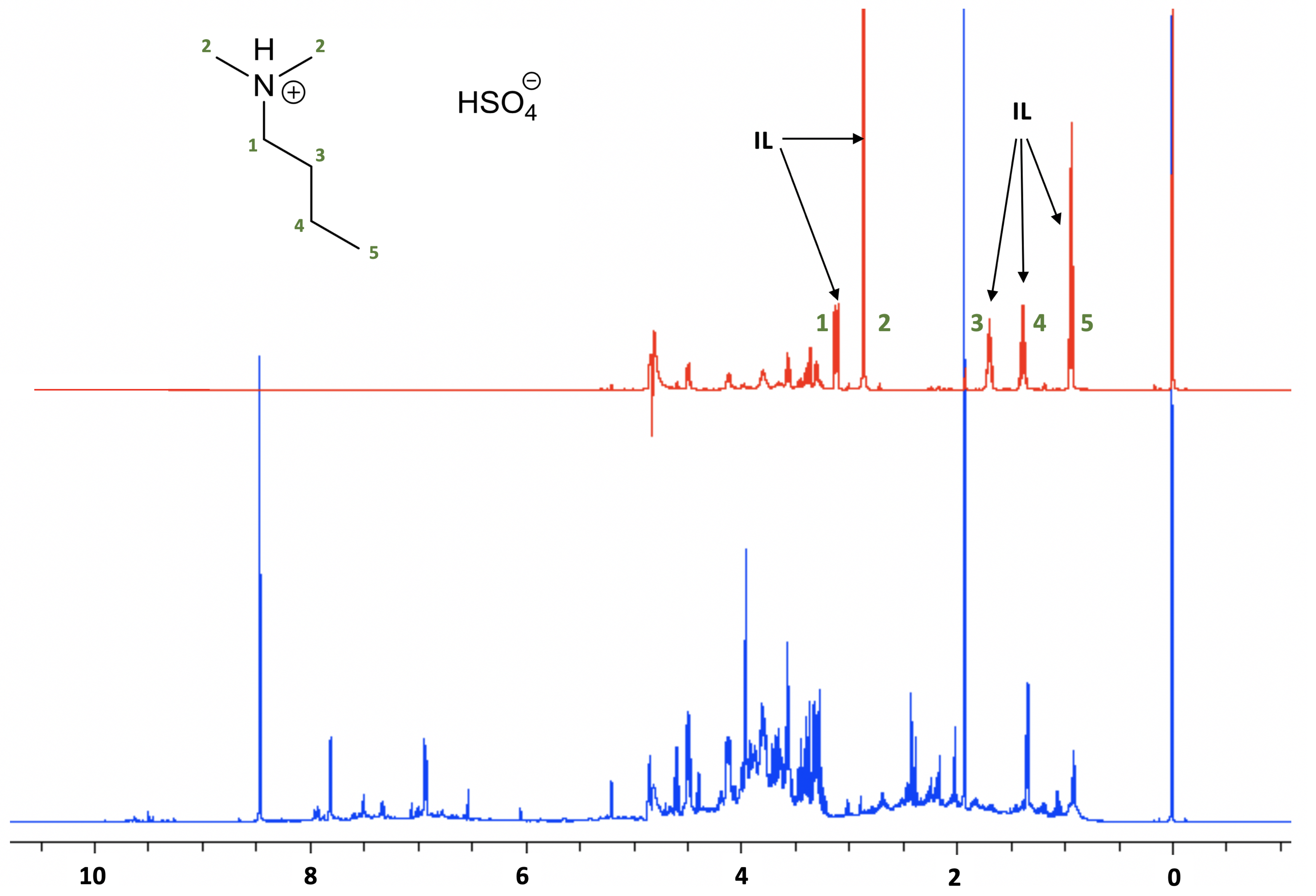

Supplement: S7 Fig — 1H-NMR spectra obtained from EFB pre-processed by ionic liquids (red) and by microwaving at 230°C (blue). The five resonances assigned to the ionic liquid are indicated on the spectrum, with the protons responsible (1–5) shown on the structure of the ionic liquid N,N dimethylbutylammonium hydrogen sulfate (top left). (TIF) [file pone.0224771.s007.tif]

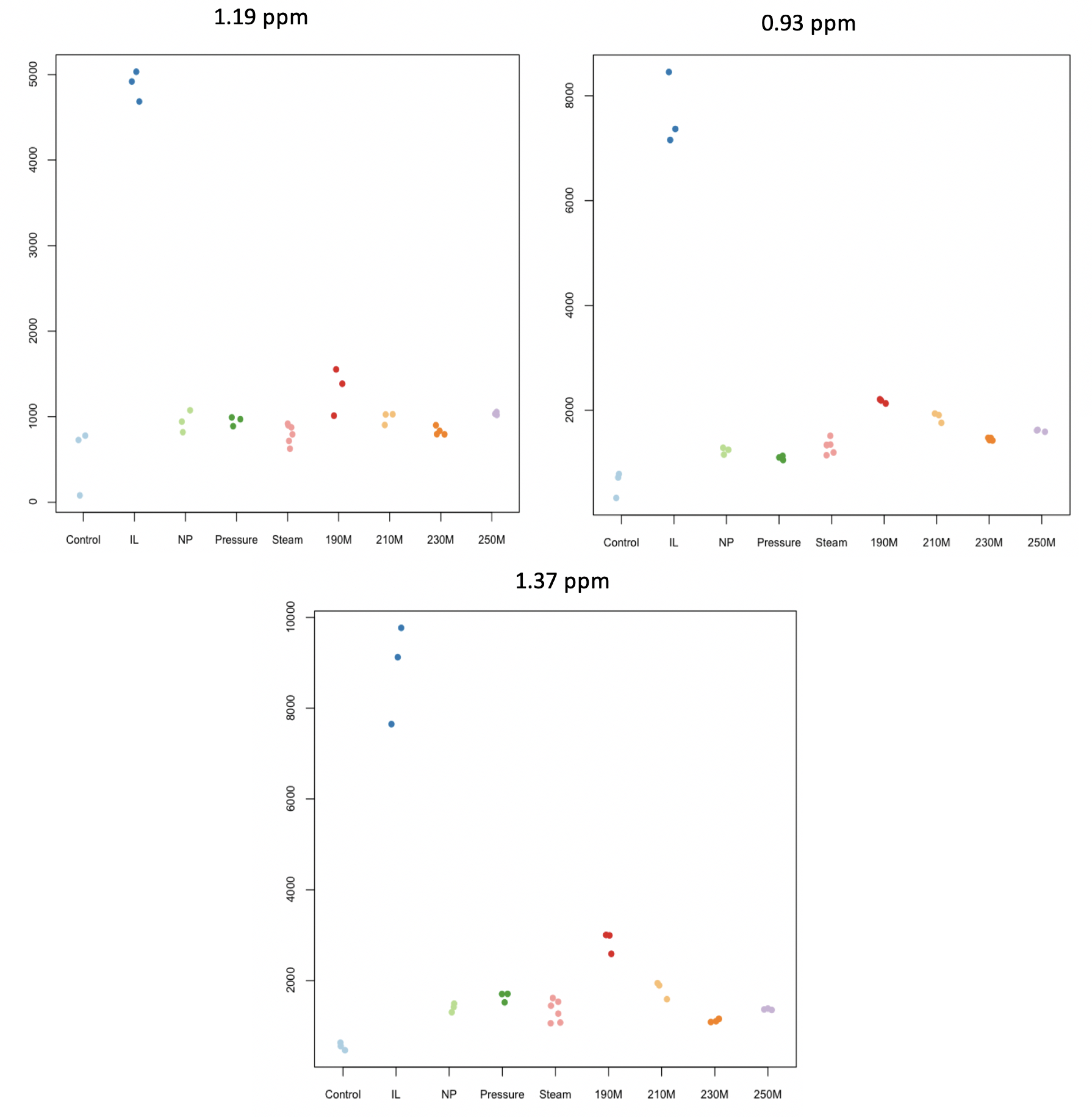

Supplement: S8 Fig — Chemical shifts (shown above plots) correspond to the structure of the ionic liquid N,N dimethylbutylammonium hydrogen sulfate. (TIF) [file pone.0224771.s008.tif]

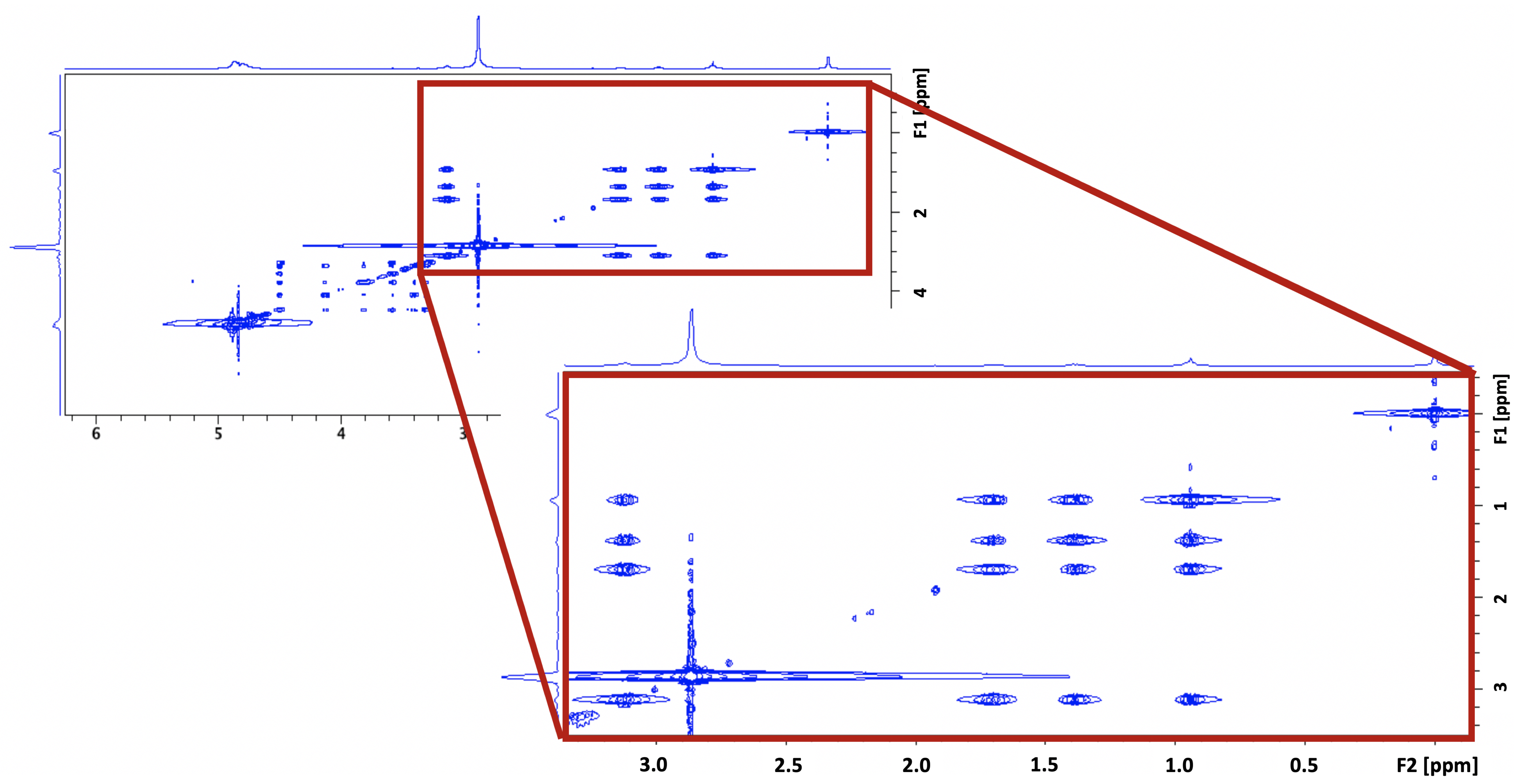

Supplement: S9 Fig — The expanded region shows the spin system from the trace ionic liquid N,N dimethylbutylammonium hydrogen sulfate present, with assignments as shown in S5 Fig. (TIF) [file pone.0224771.s009.tif]

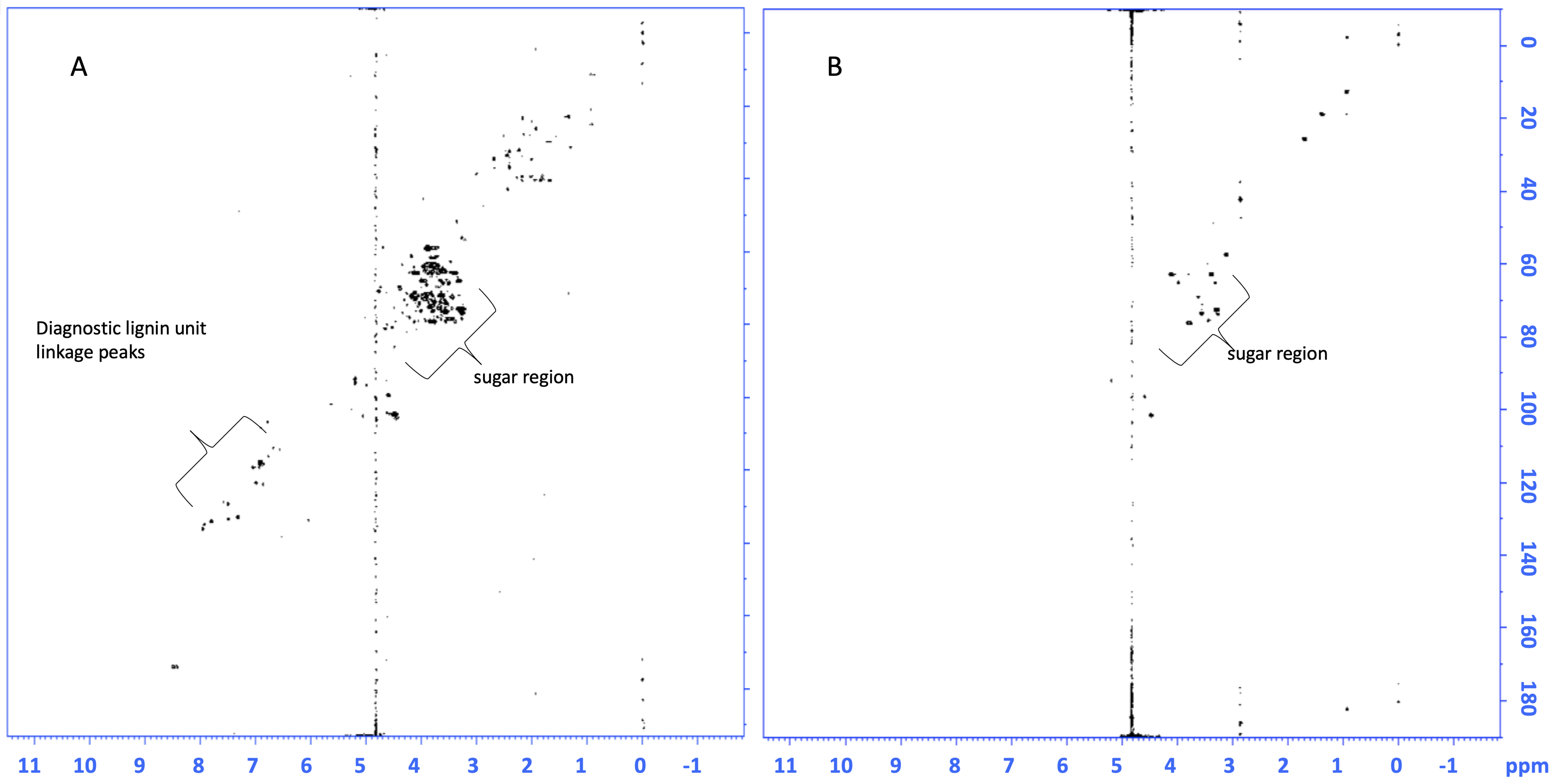

Supplement: S10 Fig — HSCQ obtained for EFB pre-processed by microwave (A) and IL (B) before anaerobic digestion. These show peaks for sugars and lignin oligomers in the microwaved sample, but not in that pre-processed by IL. (TIF) [file pone.0224771.s010.tif]

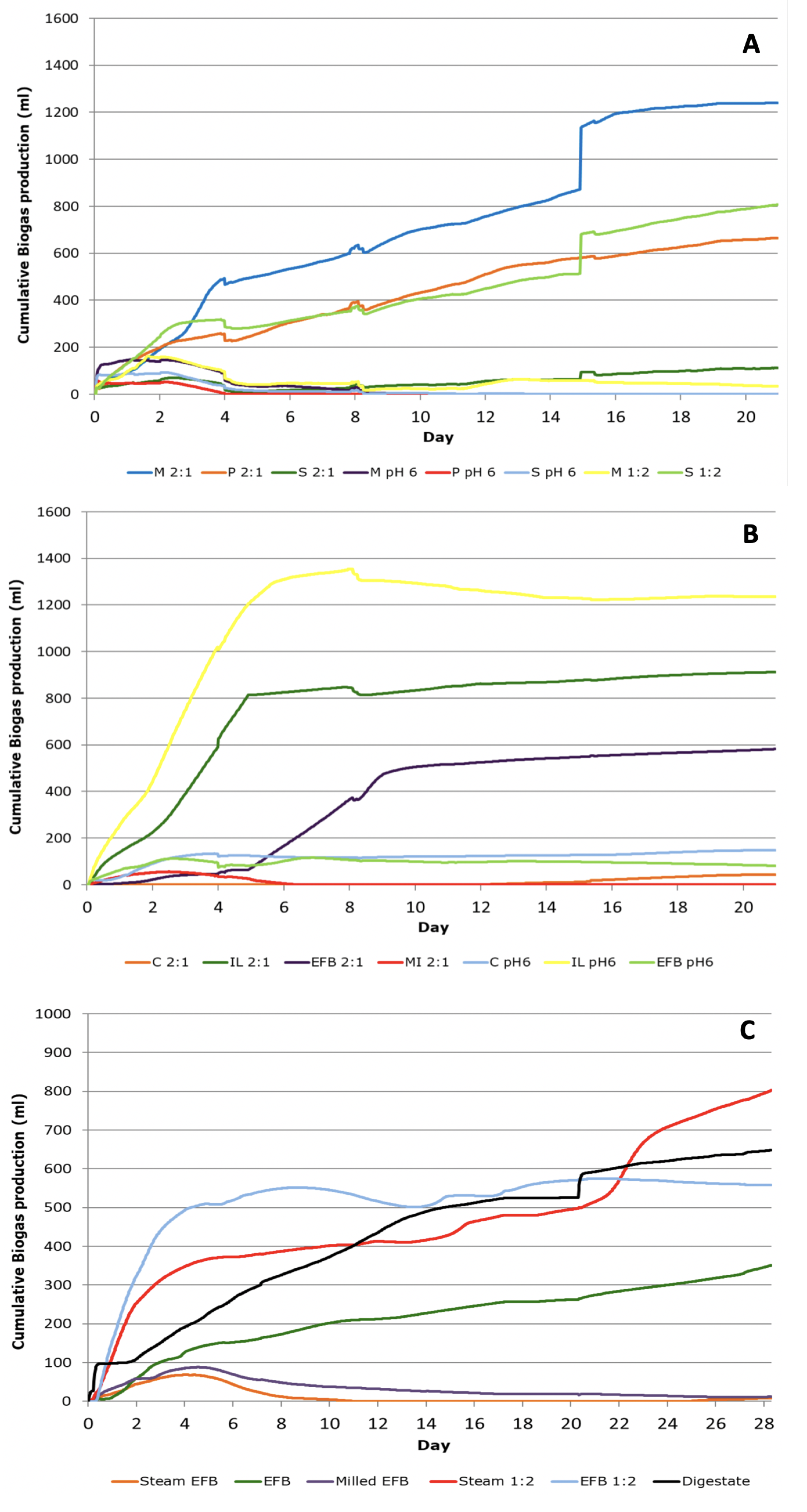

Supplement: S11 Fig — In run 1 (A), an increase in biogas production is seen at day 15, when one sample was removed for feeding trials, suggesting that this sample was producing biogas at a slower rate than the remaining two. Key: M = microwave, P = Pressure, S = Steam, C = Compost, IL = Ionic Liquid, EFB = Untreated EFB (no pre-processing), MI = Milled. Where digestate:EFB ratios are given (2:1 or 1:2), AD was carried out at pH7; where pH6 is shown, the ratio is 2:1. (TIF) [file pone.0224771.s011.tif]

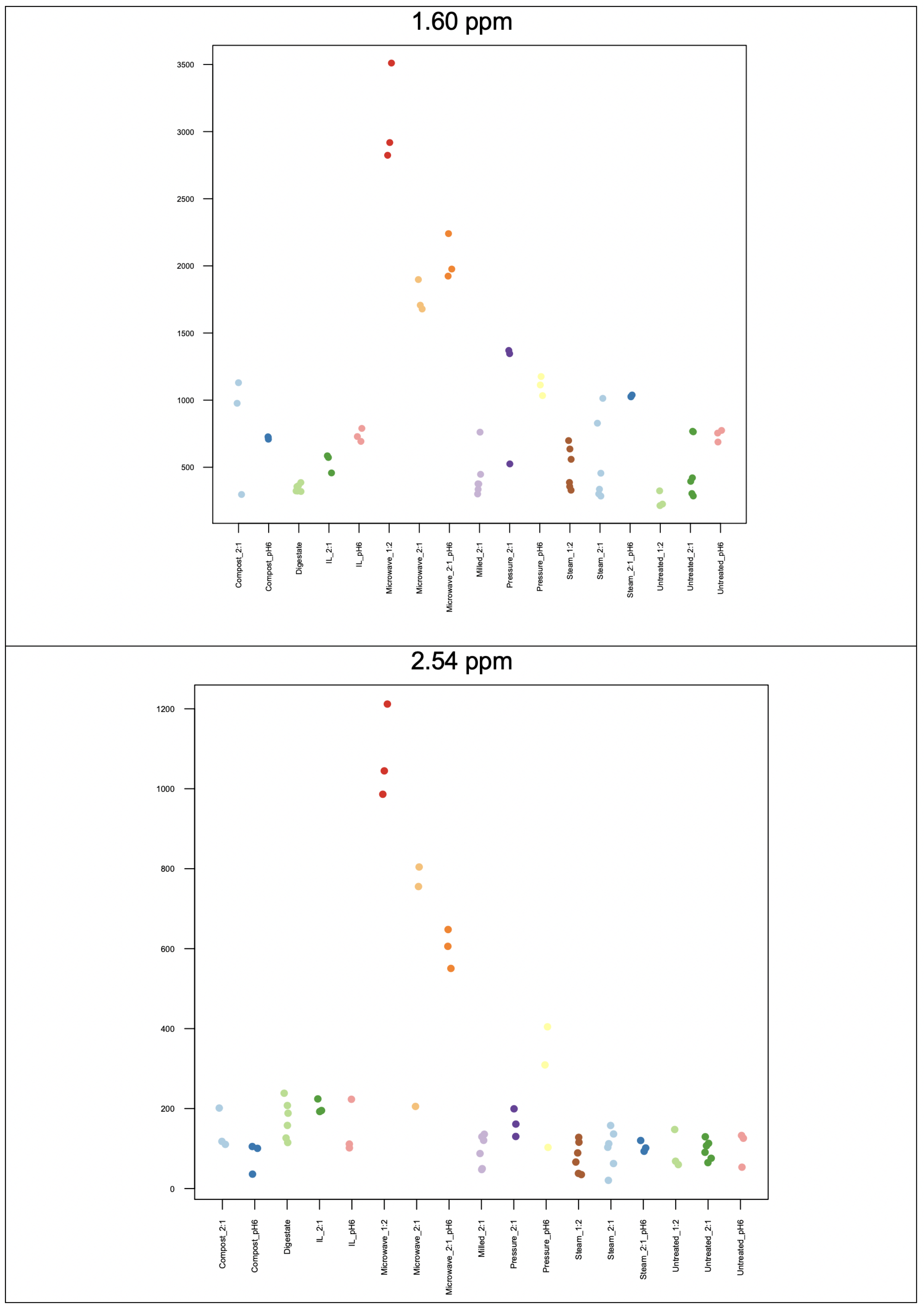

Supplement: S12 Fig — Chemical shifts (shown above plots) were attributed to lignin dimers. (TIF) [file pone.0224771.s012.tif]

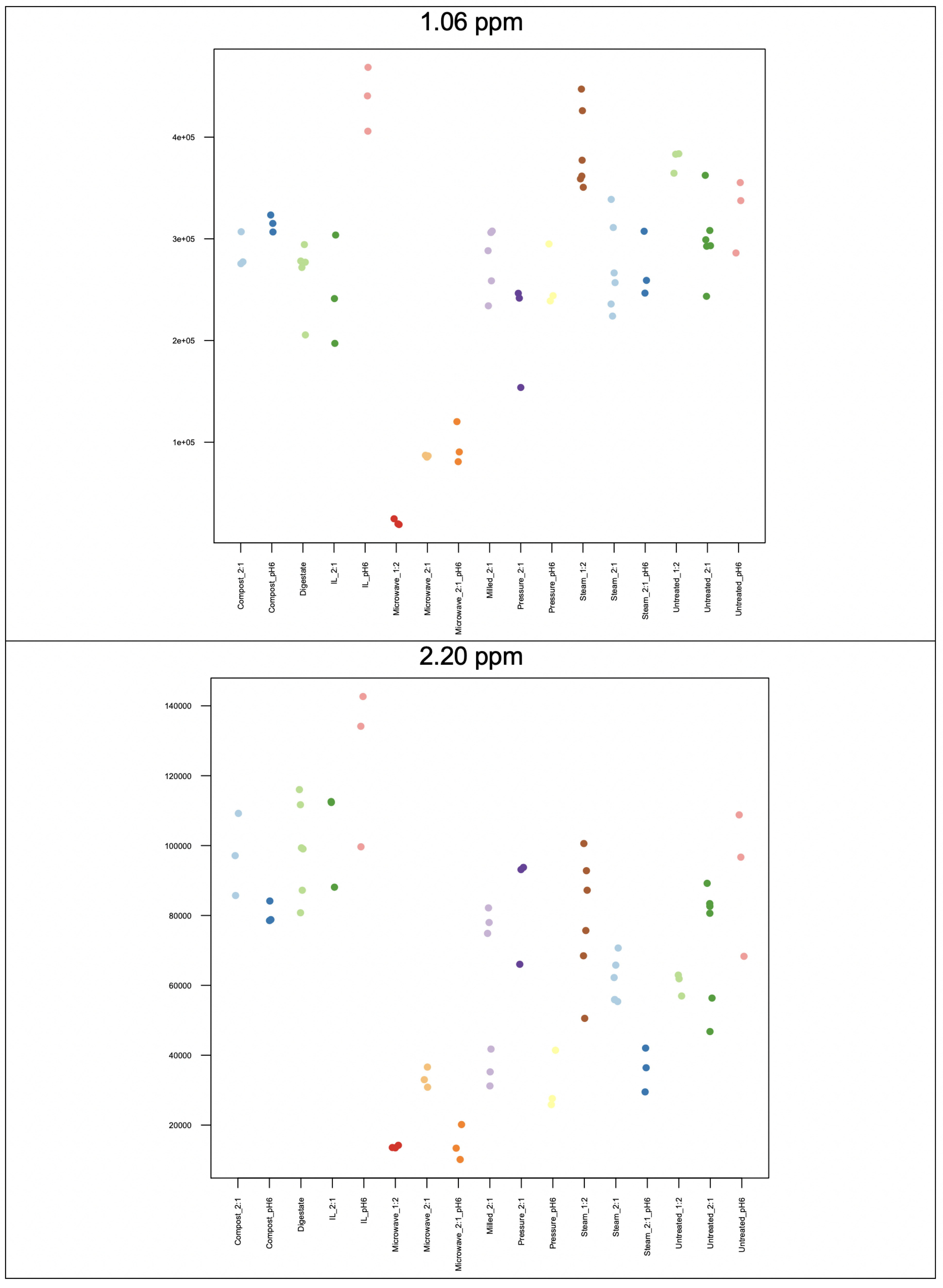

Supplement: S13 Fig — Chemical shifts (shown above plots) were matched to fragrances/flavours, possibly eucalyptol and fenchol. (TIF) [file pone.0224771.s013.tif]

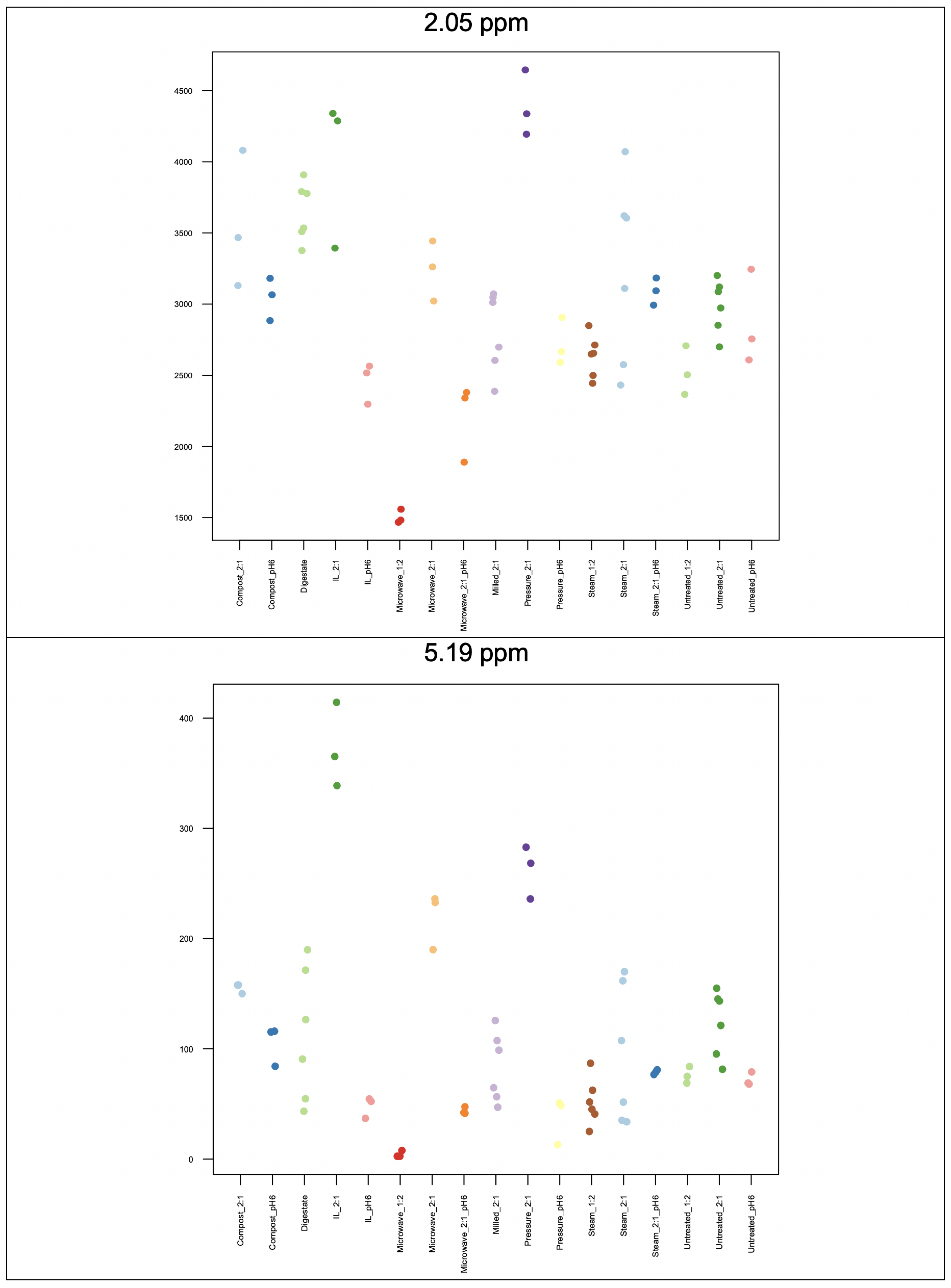

Supplement: S14 Fig — When pre-processed by ionic liquid, pressure cooking or microwaving, intensities for samples undergoing AD at pH6 are lower than for samples undergoing AD at pH7 (both at 2:1 digestate:EFB ratio). (TIF) [file pone.0224771.s014.tif]

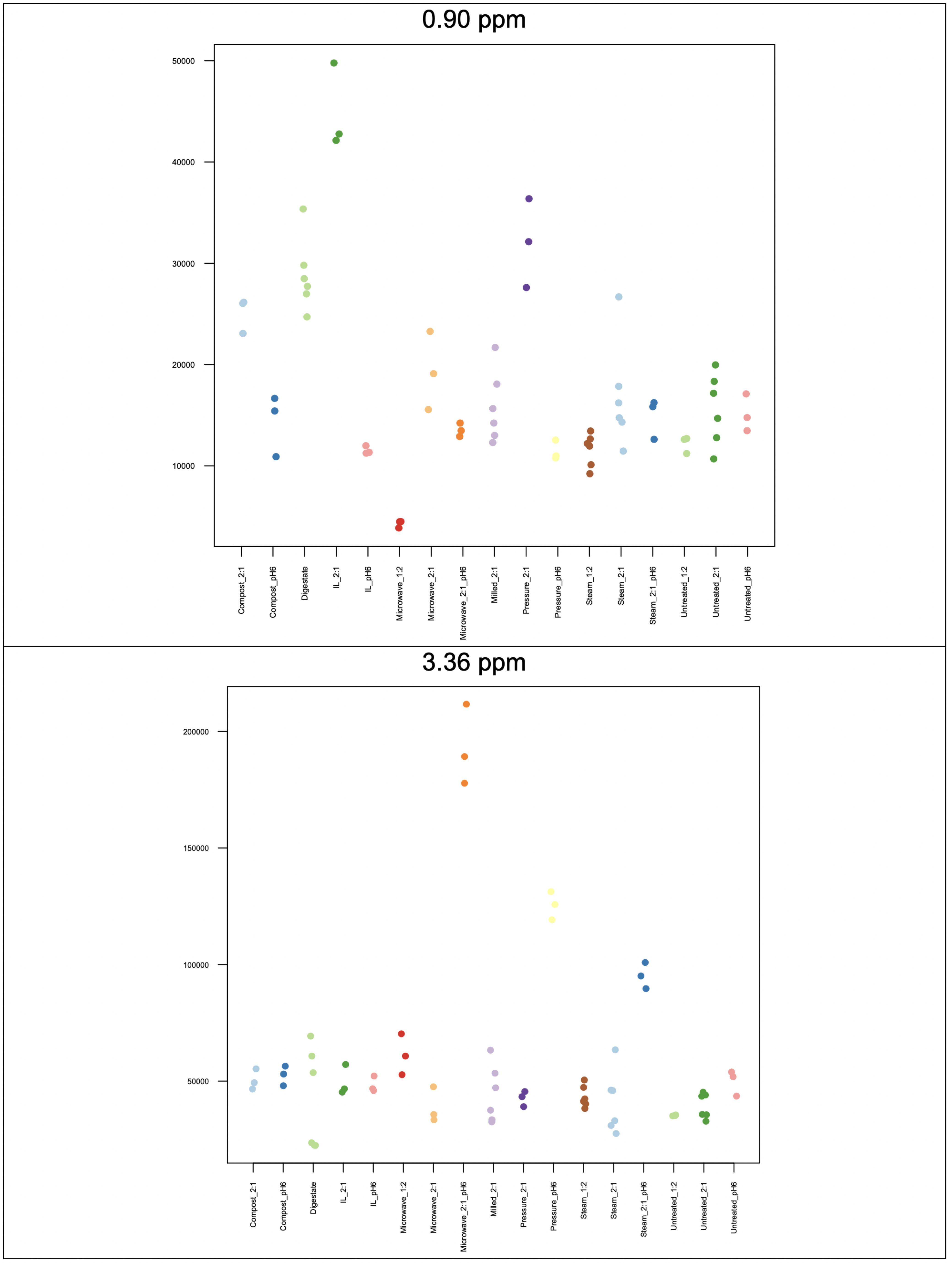

Supplement: S15 Fig — For the peak at 0.9 ppm, when pre-processed by ionic liquid or pressure cooking, samples undergoing AD at pH6 are much lower than for samples undergoing AD at pH7 (both at 2:1 digestate:EFB ratio). For the peak at 3.36 ppm, intensities are higher for samples pre-processed by microwaving, pressure cooking or steaming and digested at pH6. (TIF) [file pone.0224771.s015.tif]

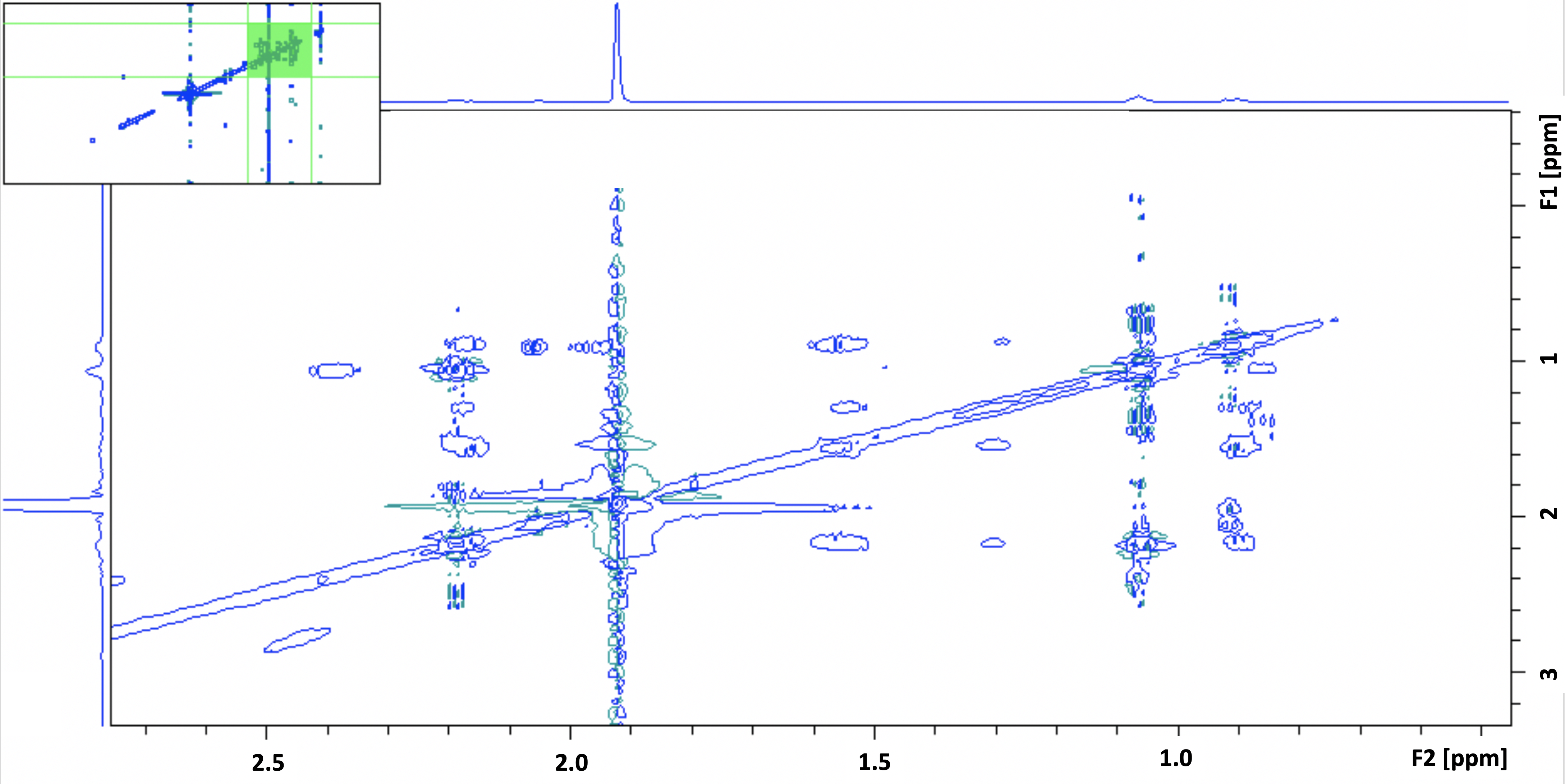

Supplement: S16 Fig — The ionic liquid, N,N-dimethylbutylammonium hydrogen sulfate, can still be seen after digestion. (TIF) [file pone.0224771.s016.tif]

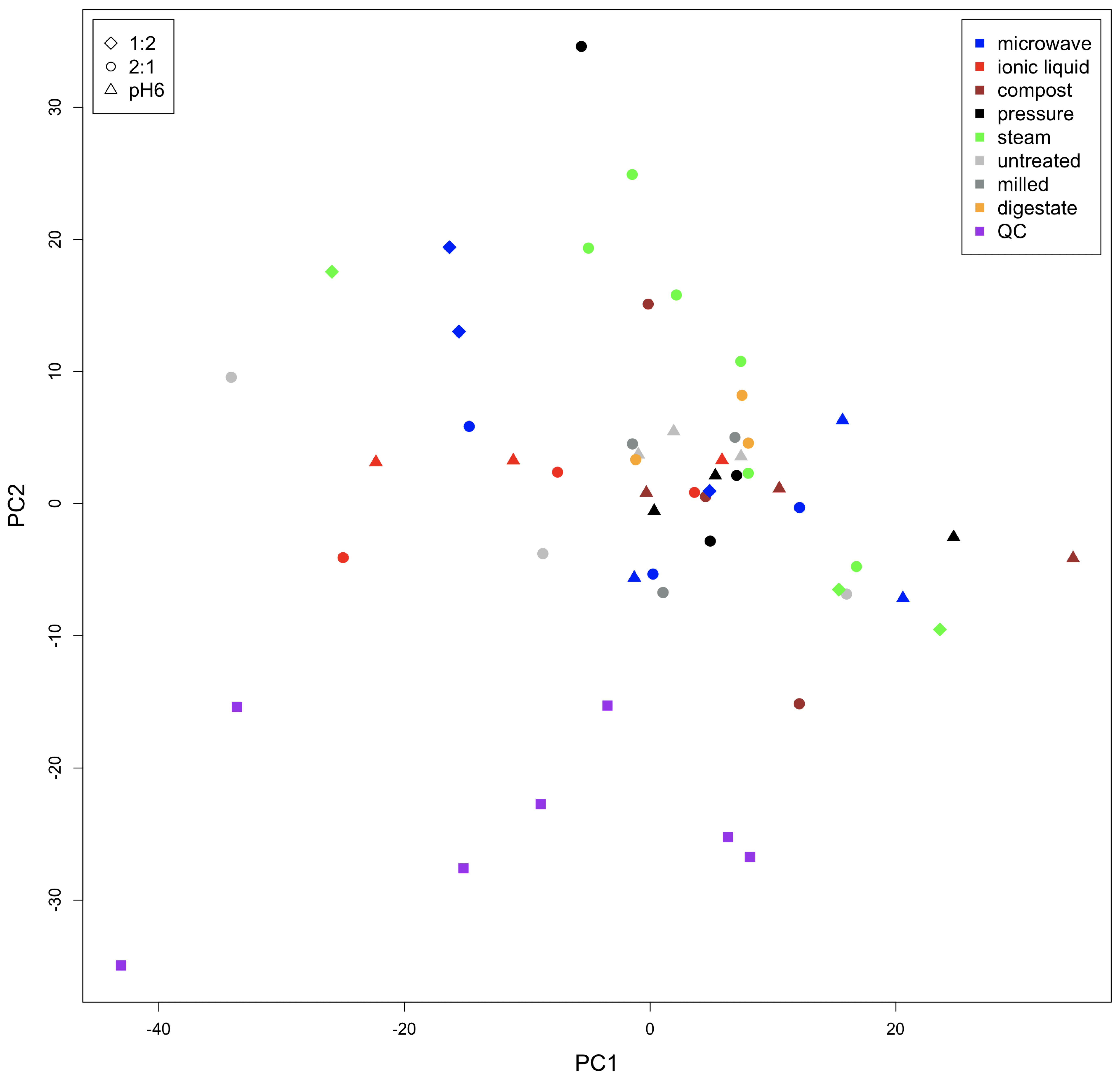

Supplement: S17 Fig — No consistent differences between pre-processing methods can be seen. (TIF) [file pone.0224771.s017.tif]

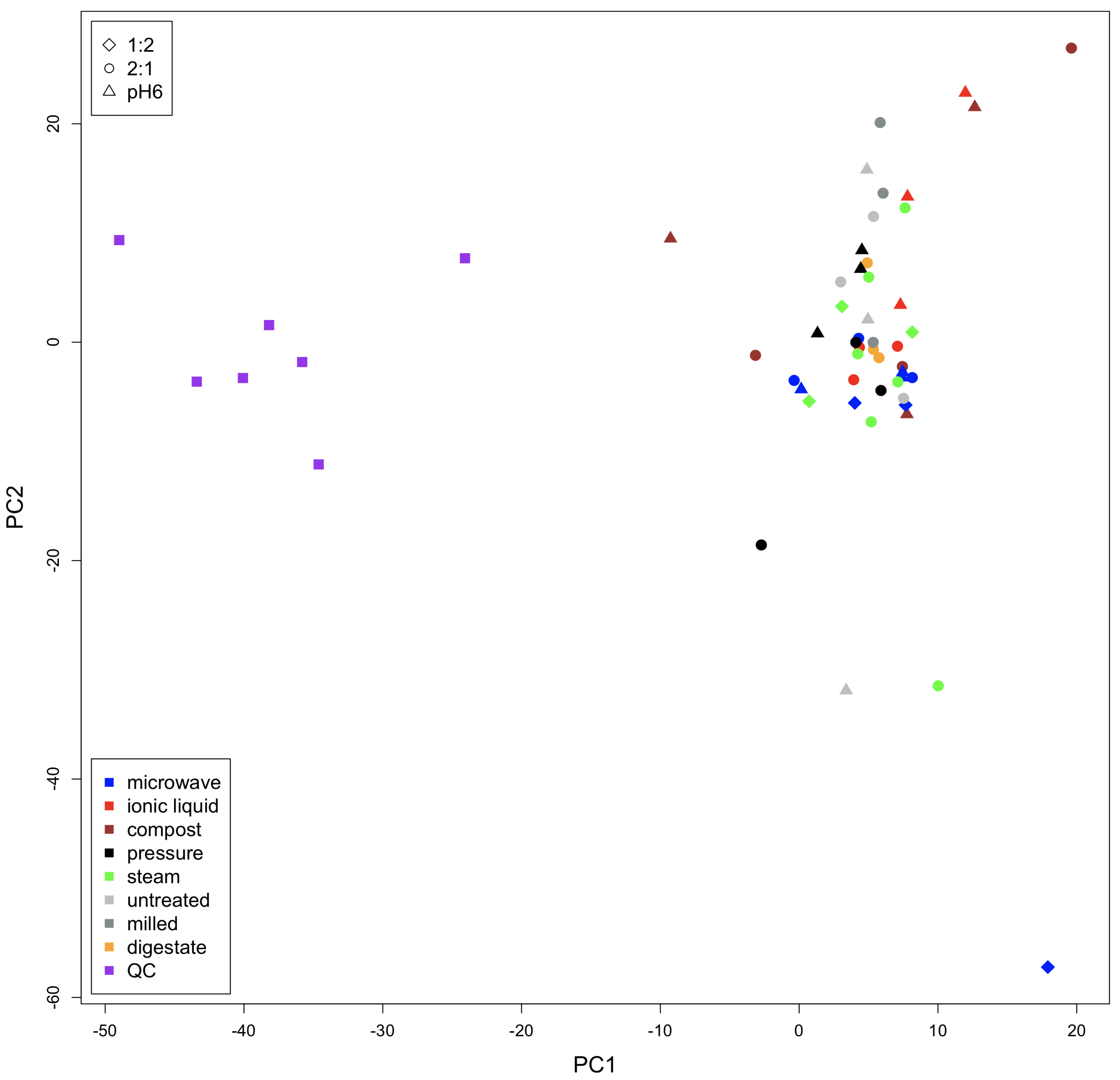

Supplement: S18 Fig — No consistent differences between pre-processing methods can be seen. (TIF) [file pone.0224771.s018.tif]

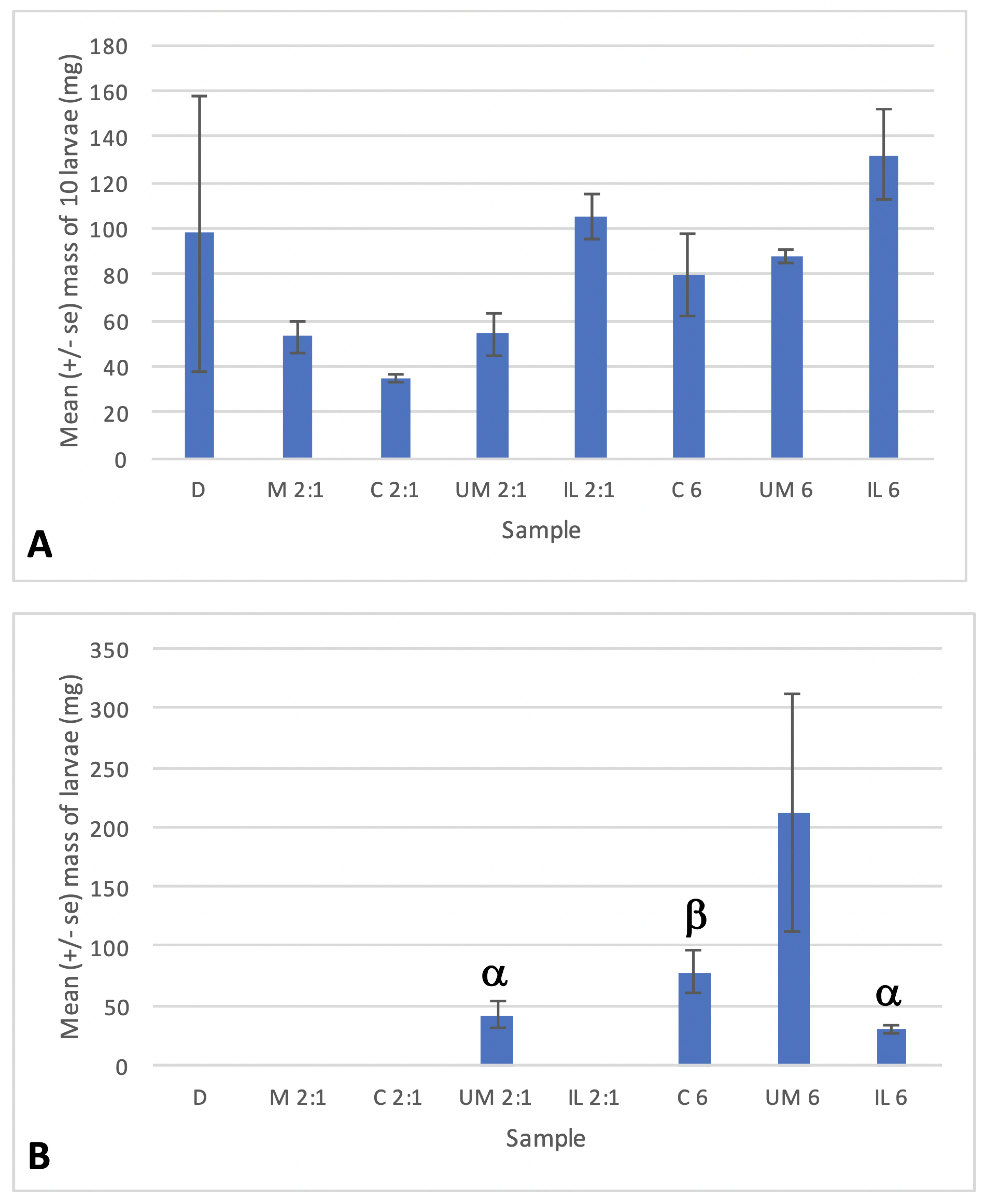

Supplement: S19 Fig — Larval mass for preliminary feeding trials at day 4 (A) and day 21 (B) of the second AD run, all 2:1 digestate:EFB ratio. Unless indicated, the average mass of ten larvae is given for each of three replicate measurements. Key: D = digestate only, M = Milled EFB, C = Composted EFB, UM = Untreated Control (not milled) EFB, IL = EFB treated with IL. AD at pH7 except for C6, UM6 and IL6 at pH6. α = only five larvae included in each replicate measurement, β = only seven-nine larvae included in each replicate measurement. (TIF) [file pone.0224771.s019.tif]
