# Supplementary material for: From waste to food: Optimising the breakdown of oil palm waste to provide substrate for insects farmed as animal feed
Source: PLoS One. 2019 Nov 7;14(11):e0224771. doi: 10.1371/journal.pone.0224771 (PMC6837394; doi:10.1371/journal.pone.0224771)
Supplement: S1 Table — *one steamed sample was removed as an outlier from this analysis. (PDF) [file pone.0224771.s020.pdf]

Data Set	Before batch correction	After batch correction
Pre-processed EFB negative mode*	2.604	0.356
Pre-processed EFB positive mode	4.059	0.455
Digested EFB negative mode	0.950	0.135
Digested EFB positive mode	2.111	0.083
