# Supplementary material for: From waste to food: Optimising the breakdown of oil palm waste to provide substrate for insects farmed as animal feed
Source: PLoS One. 2019 Nov 7;14(11):e0224771. doi: 10.1371/journal.pone.0224771 (PMC6837394; doi:10.1371/journal.pone.0224771)
Supplement: S2 Table — (PDF) [file pone.0224771.s021.pdf]

Run	Sample type	BMP Volatile Solids Ratio (Digestate VS: EFB VS)	pH	Feedstock added (g)	5M acetic acid added (ml)
1	Microwave EFB	2:1	7	64.35	-
1	Pressure cooked EFB	2:1	7	7.69	-
1<br>3	Steamed EFB	2:1	7	8.13<br>5.66	-
1	Microwave EFB	2:1	6	64.35	4.2
1	Pressure cooked EFB	2:1	6	7.69	5
1	Steamed EFB	2:1	6	8.13	5.1
1	Microwave EFB	1:2	7	257.4	-
1<br>3	Steamed EFB	1:2	7	32.51<br>22.64	-
3	EFB	1:2	7	12.59	-
2	Composted EFB	2:1	7	5.18	-
2	Ionic liquid EFB	2:1	7	28.89	-
2<br>3	EFB	2:1	7	3.15<br>3.15	-
2<br>3	Milled EFB	2:1	7	2.98<br>3.03	-
2	Composted EFB	2:1	6	5.18	4
2	Ionic liquid EFB	2:1	6	28.89	4
2	Untreated EFB	2:1	6	3.15	4
2<br>3	Digestate only	-	7	-	-
