# Supplementary material for: From waste to food: Optimising the breakdown of oil palm waste to provide substrate for insects farmed as animal feed
Source: PLoS One. 2019 Nov 7;14(11):e0224771. doi: 10.1371/journal.pone.0224771 (PMC6837394; doi:10.1371/journal.pone.0224771)
Supplement: S3 Table — (PDF) [file pone.0224771.s022.pdf]

Pre-processing method	VFA (gL^{-1})
Microwaved 2:1	1.20
Pressure cooked 2:1	2.20
Steamed 2:1	2.10
Microwaved 2:1 pH6	10.55
Pressure cooked 2:1 pH6	20.21
Steamed 2:1 pH6	30.67
Microwaved 1:2	3.67
Steamed 1:2	4.78
Composted 2:1	2.95
Ionic Liquid 2:1	1.00
Untreated EFB 2:1	3.75
Milled 2:1	0.95
Composted 2:1 pH6	10.82
Ionic Liquid 2:1 pH6	6.97
Untreated EFB 2:1 pH6	8.62
Digestate only	1.26
